# The Cost-Effectiveness of Low-Cost Essential Antihypertensive Medicines for Hypertension Control in China: A Modelling Study

**DOI:** 10.1371/journal.pmed.1001860

**Published:** 2015-08-04

**Authors:** Dongfeng Gu, Jiang He, Pamela G. Coxson, Petra W. Rasmussen, Chen Huang, Anusorn Thanataveerat, Keane Y. Tzong, Juyang Xiong, Miao Wang, Dong Zhao, Lee Goldman, Andrew E. Moran

**Affiliations:** 1 Department of Epidemiology, Fuwai Hospital, Chinese Academy of Medical Sciences and Peking Union Medical College, Beijing, China; 2 National Center for Cardiovascular Diseases, Beijing, China; 3 Department of Epidemiology, Tulane University School of Public Health and Tropical Medicine, New Orleans, Louisiana, United States of America; 4 Department of Medicine, Tulane University School of Medicine, New Orleans, Louisiana, United States of America; 5 Division of General Medicine, University of California at San Francisco, San Francisco, California, United States of America; 6 Division of General Medicine, Columbia University Medical Center, New York, New York, United States of America; 7 School of Medicine and Health Management, Tongji Medical College, Huazhong University of Science and Technology, Wuhan, China; 8 Department of Epidemiology, Capital Medical University Beijing Anzhen Hospital and Beijing Institute of Heart, Lung and Blood Vessel Diseases, Beijing, China; 9 Columbia University College of Physicians and Surgeons, New York, New York, United States of America; Stanford University, UNITED STATES

## Abstract

**Background:**

Hypertension is China’s leading cardiovascular disease risk factor. Improved hypertension control in China would result in result in enormous health gains in the world’s largest population. A computer simulation model projected the cost-effectiveness of hypertension treatment in Chinese adults, assuming a range of essential medicines list drug costs.

**Methods and Findings:**

The Cardiovascular Disease Policy Model-China, a Markov-style computer simulation model, simulated hypertension screening, essential medicines program implementation, hypertension control program administration, drug treatment and monitoring costs, disease-related costs, and quality-adjusted life years (QALYs) gained by preventing cardiovascular disease or lost because of drug side effects in untreated hypertensive adults aged 35–84 y over 2015–2025. Cost-effectiveness was assessed in cardiovascular disease patients (secondary prevention) and for two blood pressure ranges in primary prevention (stage one, 140–159/90–99 mm Hg; stage two, ≥160/≥100 mm Hg). Treatment of isolated systolic hypertension and combined systolic and diastolic hypertension were modeled as a reduction in systolic blood pressure; treatment of isolated diastolic hypertension was modeled as a reduction in diastolic blood pressure. One-way and probabilistic sensitivity analyses explored ranges of antihypertensive drug effectiveness and costs, monitoring frequency, medication adherence, side effect severity, background hypertension prevalence, antihypertensive medication treatment, case fatality, incidence and prevalence, and cardiovascular disease treatment costs. Median antihypertensive costs from Shanghai and Yunnan province were entered into the model in order to estimate the effects of very low and high drug prices. Incremental cost-effectiveness ratios less than the per capita gross domestic product of China (11,900 international dollars [Int$] in 2015) were considered cost-effective. Treating hypertensive adults with prior cardiovascular disease for secondary prevention was projected to be cost saving in the main simulation and 100% of probabilistic simulation results. Treating all hypertension for primary and secondary prevention would prevent about 800,000 cardiovascular disease events annually (95% uncertainty interval, 0.6 to 1.0 million) and was borderline cost-effective incremental to treating only cardiovascular disease and stage two patients (2015 Int$13,000 per QALY gained [95% uncertainty interval, Int$10,000 to Int$18,000]). Of all one-way sensitivity analyses, assuming adherence to taking medications as low as 25%, high Shanghai drug costs, or low medication efficacy led to the most unfavorable results (treating all hypertension, about Int$47,000, Int$37,000, and Int$27,000 per QALY were gained, respectively). The strengths of this study were the use of a recent Chinese national health survey, vital statistics, health care costs, and cohort study outcomes data as model inputs and reliance on clinical-trial-based estimates of coronary heart disease and stroke risk reduction due to antihypertensive medication treatment. The limitations of the study were the use of several sources of data, limited clinical trial evidence for medication effectiveness and harms in the youngest and oldest age groups, lack of information about geographic and ethnic subgroups, lack of specific information about indirect costs borne by patients, and uncertainty about the future epidemiology of cardiovascular diseases in China.

**Conclusions:**

Expanded hypertension treatment has the potential to prevent about 800,000 cardiovascular disease events annually and be borderline cost-effective in China, provided low-cost essential antihypertensive medicines programs can be implemented.

## Abstract 2

In a Markov-style simulation model, Andrew Moran and colleagues estimate the reduction in cardiovascular disease and cost-effectiveness of broad provision of antihypertensive medications in China.

## Editors' Summary

**Background:**

Worldwide, in 2008, more than one billion people had high blood pressure (hypertension), a condition that is responsible for about 10 million deaths annually from heart attacks, stroke, and other cardiovascular diseases (CVDs). Hypertension, which rarely has any symptoms, is diagnosed by measuring blood pressure (BP), the force that blood circulating in the body exerts on the inside of large blood vessels. BP is highest when the heart contracts to pump blood out (systolic BP) and lowest when the heart relaxes and refills (diastolic BP). Normal adult BP is defined as a systolic BP of less than 120 millimeters of mercury (mm Hg) and a diastolic BP of less than 80 mm Hg (a BP of <120/80 mm Hg). A BP of 140–159/90–99 mm Hg indicates mild (stage one) hypertension; a BP of ≥160/≥100 mg Hg indicates severe (stage two) hypertension. Many factors affect BP, but overweight people and individuals who eat fatty or salty food are at high risk of developing hypertension. Lifestyle changes and/or antihypertensive drugs can be used to control the condition.

**Why Was This Study Done?:**

Hypertension is the leading cardiovascular risk factor in China, the world’s most populous country. About 325 million adults in China have hypertension, but less than half are aware of their condition, only 34% of Chinese adults with hypertension are treated with antihypertensive drugs, and only 28% of treated individuals achieve a BP of <140/90 mm Hg. Improved hypertension control would yield enormous health gains in China, but would these gains outweigh the costs of this intervention? The World Health Organization defines a “highly cost-effective” intervention as one for which the incremental cost effectiveness ratio (ICER; in this case, the ratio of the cost difference between the intervention and no intervention to the difference in outcomes) is less than a country’s gross domestic product (GDP) per capita (a country’s total economic output divided by its number of inhabitants) per quality-adjusted life year gained (a QALY is a measure of disease burden that considers both the quality and quantity of life lived). Here, the researchers use a computer simulation model to project the cost-effectiveness of hypertension treatment in Chinese adults using the low-cost antihypertensive drugs included on the national essential medicines list. In China, most patients pay for drugs out-of-pocket, but several antihypertensive medications with affordable prices are available in government-sponsored primary health facilities.

**What Did the Researchers Do and Find?:**

The researchers used a computer model called the “Cardiovascular Disease Policy Model-China” to simulate the costs of hypertension screening, essential medicines program implementation, hypertension control program administration, drug treatment and monitoring, and the QALYs gained by preventing CVD in Chinese adults with untreated hypertension aged 35–84 y between 2015 and 2025. According to the model, treating hypertension for both primary prevention of CVD (reduction of hypertension in healthy individuals to prevent the development of CVD) and secondary prevention (reduction of hypertension in people who already have CVD to prevent further heart attacks or strokes) would prevent between 600,000 and a million CVD events annually. Treating only patients with CVD and patients with severe hypertension was borderline cost-effective. The ICER of this intervention was between Int$10,000 and Int$18,000 per QALY gained; China’s GDP per capita is Int$11,900.

**What Do These Findings Mean?:**

These findings suggest that an expanded program of treatment for hypertension could prevent about 800,000 cardiovascular events every year in China. Such a program should be borderline cost-effective, provided low-cost essential antihypertensive drugs are used to control hypertension. As with all computer simulation studies, the numerous assumptions incorporated into the model limit the accuracy of these findings. For example, some model inputs were derived from studies of non-Chinese patients and may not accurately represent the Chinese population. Moreover, the model only considers the cost-effectiveness of using medications to control hypertension and does not consider the potential effects of lifestyle changes. Importantly, additional simulations indicate that the cost-effectiveness of the intervention would be greatly reduced if adherence to treatment were lowered or drug costs were increased. Thus, full implementation of the essential medicinesprogram and subsidized drug costs program will be needed to reap the full benefits of improved hypertension control in China.

**Additional Information:**

This list of resources contains links that can be accessed when viewing the PDF on a device or via the online version of the article at http://dx.doi.org/10.1371/journal.pmed.1001860.

- The US National Heart Lung and Blood Institute has patient information about high BP (in English and Spanish) and a guide to lowering BP through diet
- The American Heart Association provides information on hypertension and on CVDs (in several languages); it also provides personal stories about dealing with high BP
- The UK National Health Service (NHS) Choices website provides detailed information for patients about hypertension (including a personal story) and about CVD
- The World Health Organization provides information on CVD and controlling hypertension; "A Global Brief on Hypertension" was publi shed on World Health Day 2013; WHO-CHOICE provides information on choosing cost-effective interventions
- MedlinePlus provides links to further information about high BP, heart disease, and stroke (in English and Spanish)

## Introduction

High blood pressure (BP) is the leading risk factor for cardiovascular disease (CVD) in China, and uncontrolled high BP is responsible for more of total disease burden in China than any other single risk factor [1]. Approximately 325 million, or about 30%, of Chinese adults aged 18 y or older have hypertension [2]. Among Chinese adults with hypertension, less than half are aware of their diagnosis, about 34% are treated with medications to lower BP, and less than 28% of those treated are controlled to a goal of <140 mm Hg systolic BP and <90 mm Hg diastolic BP [2]. Though the potential health gains from hypertension control would be enormous, the cost-effectiveness of implementing Chinese BP treatment guidelines has not been assessed.

China’s 2009 national health reform expanded health insurance coverage dramatically, but most patients still pay for outpatient clinic visits and medications out-of-pocket [3–5]. The 2009 reforms introduced a list of “essential” antihypertensive medications with fixed, affordable prices required in government-sponsored primary health facilities [6]. Negotiation for and enforcement of lower drug costs is done at the provincial or municipal level, and enforcement of the “zero profit” rule has not been uniform across the health system [7]. We used the CVD Policy Model-China, a national scale computer simulation model [8,9], to assess the cost-effectiveness of treating hypertension in China, using low-cost medications on the national essential medicines list.

## Methods

### CVD Policy Model-China Overview

**Fig 1:**
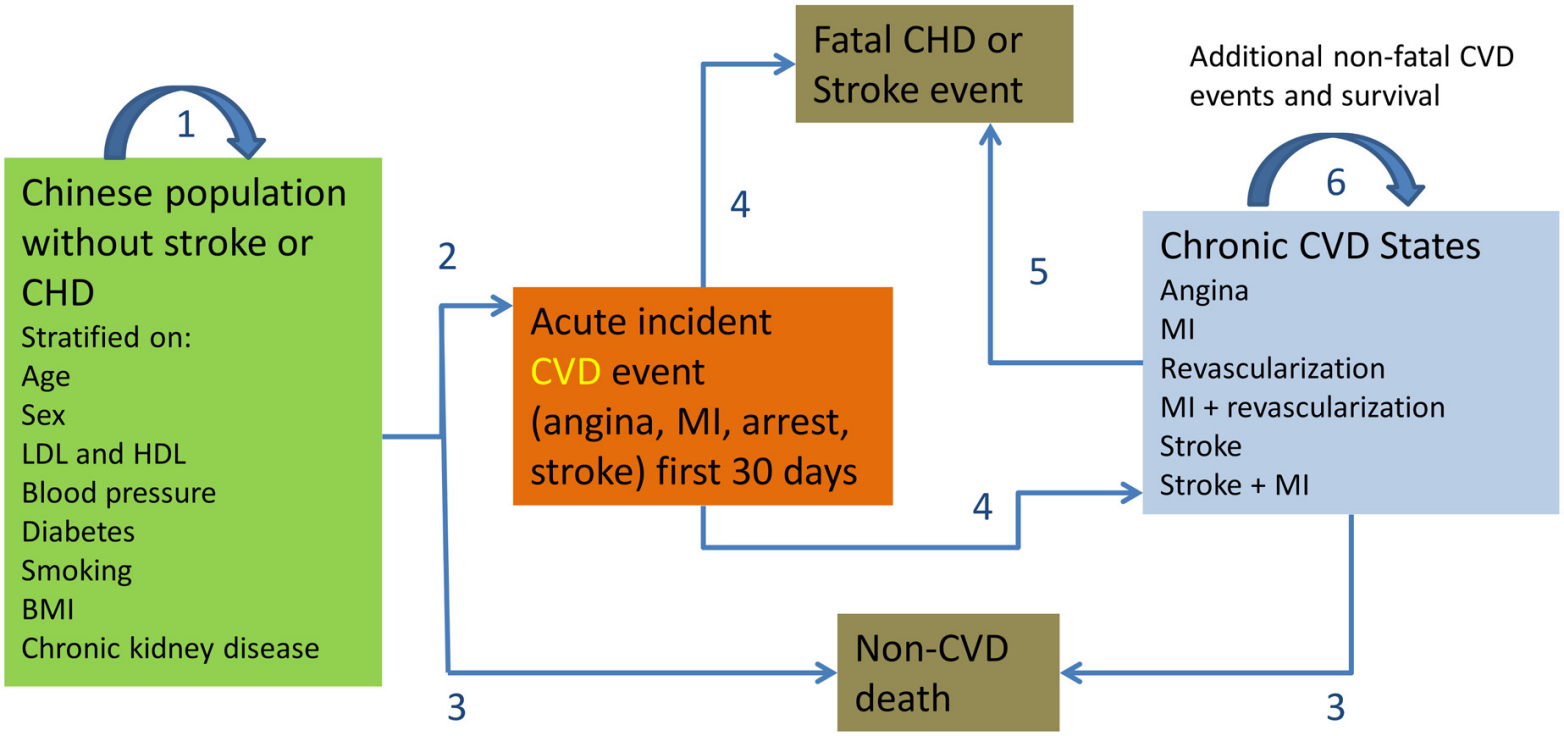
CVD Policy Model-China structure. State transitions are numbered in the diagram. Transition 1 = remain in CVD-free state. Transition 2 = incident CVD. Transition 3 = non-CVD death. Transitions 4 and 5 = survival or case fatality. Transition 6 = survival with or without repeat CVD event in chronic CVD patients.

Population-based mathematical simulation models are appropriate for estimating the average value of implementing national clinical practice guidelines. The CVD Policy Model-China is a computer-simulation, state-transition (Markov cohort) mathematical model of coronary heart disease and stroke incidence, prevalence, mortality, non-CVD deaths, and health care costs at the population level in adults aged 35–84 y in China (Fig 1, S1 Text) [8]. The model predicts annual coronary heart disease and stroke incidence and non-CVD mortality among persons without CVD, stratified into cells by age, sex, systolic BP, high-density lipoprotein (HDL) and low-density lipoprotein (LDL) cholesterol levels, body mass index (BMI), and status of isolated diastolic hypertension, hypertension treatment, chronic kidney disease, smoking, and diabetes mellitus. Simulations projecting CVD in future years incorporate demographic changes and preserve age-related trends in risk factors, event rates, and case fatality. Appropriate to the diagnostic definitions of CVDs, the model assumes that survivors persist in a chronic disease state (linear model without remission). The model also predicts life years, coronary heart disease and stroke events, CVD mortality, and non-CVD mortality among patients with CVD. Each policy model health state and event has an annual cost and quality-of-life adjustment.

National prevalence, joint distributions, and means of risk factors were estimated from the International Collaborative Study of Cardiovascular disease in Asia (InterASIA). This included proportions of Chinese adults with systolic BP of <130, 130–139, 140–159, and ≥160 mm Hg and diastolic BP of <80, 80–89, 90–99, and ≥100 mm Hg, and the proportion with self-reported untreated hypertension. Systolic BP of 140–159 mm Hg and ≥160 mm Hg correspond in both Chinese Society of Hypertension guidelines [10] and European Society of Cardiology guidelines [11] to stage one and ≥stage two hypertension. Isolated diastolic hypertension was categorized into two corresponding categories: diastolic of 90–99 or ≥100 mm Hg, both accompanied by systolic BP of <140 mm Hg. For treatment of isolated systolic hypertension and combined systolic and diastolic hypertension, we simulated a reduction in systolic BP; for treatment of isolated diastolic hypertension, we simulated a reduction in diastolic BP. All of these subcategories of hypertension received the intervention in treatment simulations.

The CVD Policy Model-China defined coronary heart disease as myocardial infarction (ICD-9 410 and 412 or ICD-10 I21 and I22), angina and other coronary heart disease (ICD-9 411, 413, and 414 or ICD-10 I20 and I23–I25), and a fixed proportion of “ill-defined” CVD-coded events and deaths (ICD-9 codes 427.1, 427.4, 427.5, 428, 429.0, 429.1, 429.2, 429.9, and 440.9 or ICD-10 I47.2, I49.0, I46, I50, I51.4, I51.5, I51.9, and I70.9) [12]. Stroke was defined by ICD-9 codes 430–438 (excluding transient ischemic attack) or ICD-10 I60–I69. Starting with coronary heart disease and stroke incidence and prevalence obtained from the China Hypertension Epidemiology Follow-up Study, the CVD Policy Model-China mortality projections were calibrated to fit with age-specific and overall coronary heart disease and stroke mortality numbers for the years 2000–2010 estimated by the World Health Organization (S1 Text).

### Risk Factor Risk Coefficients and Model Calibration

Risk factor beta coefficients for LDL, HDL, diabetes, chronic kidney disease, and smoking conditioned on age and sex were estimated from the China Multi-provincial Cohort Study (CMCS) [13] using three distinct competing risk Cox proportional hazard models with coronary heart disease, total stroke, and other-cause death as the outcomes.

We assumed CVD risk reduction is due to BP reduction [14] and that BP is lowered to a similar extent across classes when comparing per-class standard doses [15,16]. We started with observational Prospective Studies Collaboration age-specific relative risks and 95% confidence intervals for coronary heart disease and stroke per 10 mm Hg change in systolic BP or 5 mm Hg diastolic BP (Table 1) [17]. Age-specific relative risk inputs were calibrated to be within ≤0.02 of these estimates and overall relative risks within 95% confidence interval bounds of the summary estimate from a large meta-analysis of randomized clinical trials of hypertension treatment (S1 Table, S2 Table) [14]. The stroke relative risk estimate was found to be close to the pooled estimate from the East Asian trials included in that analysis (0.59 [0.49–0.71], S1 Text). The resulting relative risk assumptions were validated for treatment of systolic BP in ages of 60–74 y by simulating the treatment and placebo groups of the Systolic Hypertension in the Elderly Program (SHEP) trial and comparing simulated relative rate ratios with those observed in the trial (S1 Text, S3 Table).

**Table 1:** Main assumptions for the cost-effectiveness analysis of China hypertension control policy.

Variable	Estimate (Measure of Uncertainty)	Sources
**Screening and monitoring**		
Hypertension screening frequency (used in sensitivity analysis of screening costs)		
Annual screenings if initial BP <130/80 mm Hg	1	China hypertension control program [10]
Twice yearly screenings if initial BP ≥130/80, <140.90 mm Hg	2	China hypertension control program [10]
Hypertension monitoring frequency (range)		
Annual monitoring visits for stage one hypertension	3 (2–5)	China hypertension control program [10]
Annual monitoring visits for stage two hypertension	5 (4–6)	China hypertension control program [10]
**Effectiveness**		
Average relative risk per 10 mm Hg reduction in systolic BP or 5 mm Hg reduction diastolic BP in patients 35–64 y old* (95% confidence interval)		Calibrated Prospective Cohorts Collaborative estimates to fit with meta-analysis of trials [14,18]
CHD	0.73 (0.70–0.77)	
Stroke	0.64 (0.59–0.69)	
Average relative risk per 10 mm Hg reduction in systolic BP or 5 mm Hg reduction diastolic BP in patients ≥65 y old* (95% confidence interval)		Calibrated Prospective Cohorts Collaborative estimates to fit with meta-analysis of trials [14,18]
CHD	0.77 (0.74–0.79)	
Stroke	0.69 (0.64–0.74)	
Systolic BP lowering effect, median change in category assuming 50% adherence, in mm Hg, (range of age- and sex-specific systolic BP changes assumed detailed in S1 Text)†		Trials meta-analysis [14]
Ages 35–64 y (target 140 mm Hg)		
Stage two hypertension (≥ 160 mm Hg, mean in category 175) 3.4 standard dose antihypertensive agents	22.7 (17.5–27.9)	
Stage one hypertension (140–159 mm Hg, mean in category 147) 1.1 standard dose antihypertensive agents	6.5 (4.1–8.9)	
Ages ≥65 y (target 150 mm Hg), regardless of diabetes/chronic kidney disease status		
Stage two hypertension (≥ 160 mm Hg, mean in category 175) 2.8 full-dose antihypertensive agents	17.8 (13.2–22.4)	
Stage one hypertension (140–159 mm Hg, mean in category 147) 0.9 full-dose antihypertensive agents	2.6 (1.5–3.7)	
Diastolic BP lowering effect in isolated diastolic hypertension (IDH), in mm Hg (range of age- and sex-specific systolic BP changes)		Trials meta-analysis [14]
Ages 35–84 y (target 90 mm Hg), no diabetes or chronic kidney disease		
Stage two IDH (normal systolic; ≥100 mm Hg diastolic BP) 3.0 full-dose antihypertensive agents	12.4 (8.7–16.1)	
Stage one IDH (normal systolic; 90–99 mm Hg diastolic BP) 2.0 full-dose antihypertensive agents	3.5 (2.5–4.6)	
**Drug side effects leading to discontinuation**		
Incidence per 100 person-years, based on one standard dose medication		
Common, managed as outpatient (1%–10% of users, 95% confidence interval)	5.20% (3.6%–6.6%)	Law 2003 [15],
Infrequent, hospitalized, nonfatal (<1% of users, range)	0.01% (0.01%-0.05%)	package insert data summarized by Lexicomp
Rare; intensive care or death (from case reports, range)	0.001% (0.00001%-0.01%)	package insert data summarized by Lexicomp
Proportion of rare events survived	0.99	
Proportion of rare events that are fatal	0.01	
Relative rate of side effects		Based on Law 2003 [15]
One-half standard dose	0.5	
One standard dose	1.0 (reference)	
Two standard doses	1.5	
Three standard doses	1.9	
Four standard doses	2.3	
Five standard doses	2.5	
**Costs per person, 2015 International dollars** ‡		
Acute hospitalization costs (mean)		China Health Statistics Yearbook, 2009 [19]
Stroke	2,620	
Angina pectoris	2,580	
Myocardial infarction, no revascularization procedure	5,540	
Myocardial infarction, with percutaneous coronary intervention	12,910	
Myocardial infarction, with coronary artery bypass graft surgery	26,410	
Chronic costs: incurred throughout the rest of year 1 (median)		Microeconomic Impact of CVD Survey (Huffman et al.) [20]
Stroke	650	
CHD	1,060	
Annual Chronic costs: incurred after year 1 (median)		
Stroke	420	
CHD	740	
Hypertension screening or monitoring visit cost (range) (in outpatient health center; 10 min [2–20 min])	16.50 (14.50–17.20)	WHO CHOICE (China) [21]
Laboratory test to monitor treatment with antihypertensive drugs (unit cost; sodium, potassium, and creatinine)	5.00	Beijing Municipal Commission of Development and Reform
Antihypertensive drug costs per year (average of median costs or average of lowest costs of thiazide diuretics, angiotensin converting enzyme inhibitors, calcium channel blockers, and beta blockers)		China Essential Medications Drug Cost List, 2009
0.5 standard dose	11.40 (7.20)	
1.0 standard dose	22.70 (14.40)	
1.5 standard doses	30.40 (21.60)	
2.0 standard doses	44.60 (30.40)	
3.0 standard doses	62.50 (40.00)	
3.5 standard doses	78.30 (50.10)	
4.0 standard doses	90.90 (57.60)	
5.0 standard doses	113.60 (71.90)	
Side effect costs		
Common, managed as outpatient (1%–10% of users)	80	
Infrequent, hospitalized, nonfatal (<1% of users)	450	
Rare; intensive care or death (from case reports)	1,570	
**Utility** ¶		GBD 2010 Study [22]
Acute CVD events (first 28 d)		
Acute stroke		
Days 1–3	0.70	
Days 4–28	0.88	
Acute myocardial infarction		
Days 1–2	0.58	
Days 3–28	0.94	
Chronic CVD states (remainder of first year, 365 d thereafter)		
Chronic, stable angina pectoris	0.91	
Myocardial infarction survivors (64% asymptomatic; 36% have heart failure symptoms [23], chronic heart failure weight = 0.90)	0.96	
Stroke survivors		
Side effects		
Common, managed as outpatient (1%–10% of users)	0.88	Clinical judgment
Infrequent, hospitalized, nonfatal (<1% of users)	0.70 (for 2 d)	0.50 = GBD 2010 Study weight for severe illness, e.g., terminal cancer, end stage renal or liver disease (survivors) [22]
Rare; intensive care or death (from case reports)	0.50 (for 2 d)	
Survivors	0.50 (for 7 d), then 0.80 for 30 d recovery	
Fatalities	0.00; Loss of life years starting at time of death	
**Adherence to prescribed medications**	40% overall, based on 50% continuation of prescribed medications, and 10% of doses missed among patients continuing (range: 25%–75% adherence in sensitivity analyses)	Persistence: trials meta-analysis [14] and the PURE study [24] missed doses frequency: Vrijens et al. [25]
**Annual discount rate for costs and quality adjusted life-years (QALYs)**	3%	Weinstein et al. [26]

*Relative risk reductions higher for higher baseline BP and lower for older ages; see S1 Text for details by age and sex category.

^†^Relative risks for pretreatment SBP of 150 mm Hg is shown for simplicity; effect size increases with higher pretreatment BP.

^‡^To convert cost input to Chinese currency, multiply by purchasing power parity (PPP) rate (in this case, 3.52). To convert to US$ using the current official exchange rate, multiply by (PPP/exchange rate), for example, 3.52/6.20, or by 5.68.

^¶^Quality-of-life adjustments, where 1.0 = perfect health.

### Hypertension Treatment Policy Simulations

BP lowering with treatment and the number of “standard dose” antihypertensive agents needed to meet target BP in untreated hypertensive patients according to pretreatment BP and age were based on a meta-analysis of BP treatment trials (S4 Table) [14]. Among adults with stage two hypertension and the highest pretreatment BP (mean 176 mm Hg systolic or 110 mm Hg diastolic), it was assumed that a small proportion have truly resistant hypertension, and the average treated BP was just above goal (143 mm Hg systolic or 92 mm Hg diastolic). Variance in BP change with antihypertensive treatment was based on standard deviations around the main BP change estimates observed in a meta-analysis [15]. In main simulations, complete discontinuation of prescribed medications in China one year after initiation was assumed to be 50%, based on medication discontinuation rates observed in China and other middle income countries that were sampled in the Prospective Urban Rural Epidemiology (PURE) study [24]. For all simulations, it was assumed that adherent patients miss about 10% of scheduled medication doses, leading to 10% lower effectiveness, but incur the full cost of the scheduled regimen [25]. Based on these assumptions, overall medication adherence was assumed to be 40% in main simulations, similar to a study by another group [27].

A status quo simulation projected total (first ever and repeat) myocardial infarction and stroke events, direct CVD costs, and QALYs for Chinese adults assuming no change in current levels of hypertension treatment over the years 2015–2025. The first intervention step simulated treatment of all untreated patients with existing CVD (secondary prevention). Subsequent simulations progressively added primary prevention treatment of the untreated population without existing CVD and in two steps: first, stage two hypertension alone and second, stage two plus stage one together. Among patients aged 65–84 y with systolic hypertension (with or without diastolic hypertension), those with stage two hypertension required an average of 2.7 standard dose medications to reach a systolic BP goal of 150 mm Hg, and those with stage one hypertension required an average of 0.9 standard dose medications to reach a goal systolic BP of 150 mm Hg (Table 1, S4 Table, and S5 Table). Among patients aged 35–64 y with systolic hypertension (with or without diastolic hypertension), those with stage two hypertension required an average of 3.4 standard dose medications to reach a goal systolic BP of 140 mm Hg, and those with stage one hypertension required an average of 1.2 standard dose medications to reach a goal systolic BP of 140 mm Hg. For patients of all ages with isolated diastolic hypertension, those with stage two isolated diastolic hypertension required an average of 3.0 standard dose medications to reach a goal diastolic BP of 90 mm Hg, and those with stage one isolated diastolic hypertension required an average of 1.0 standard dose medications to reach a goal diastolic BP of 90 mm Hg. Based on equivocal clinical trial evidence to support a lower target for patients with diabetes or chronic kidney disease and consequent changes to recent international guidelines, we assumed the same diagnostic thresholds and targets for patients with or without these conditions [11,28].

Median costs of drugs in four standard antihypertensive drug classes (thiazide diuretic, angiotensin converting enzyme inhibitor, beta blocker, and long-acting calcium channel blocker) in China’s 2009 national essential medicines list were averaged and inflated to 2015 (S1 Text). Combinations of standard dose medications (1.5, 2.0, 2.5, 3.0, 3.5, and 4.0 standard doses) were assigned the cumulative cost of the individual agents because the essential medicines list did not include combination agents priced lower than the cost of two separate drugs. Rates of adverse events from medication side effects were based on a meta-analysis of treatment trial side effect rates for more common events [15] and postmarketing reports for rarer events. Adverse event rates were translated into quality of life impairments, and added costs related to events ranging from transient symptoms accompanied by an office visit (common) to death (rare).

In addition to individual patient treatment costs, we simulated national hypertension control program costs. China’s central government recently financed opportunistic hypertension screening in adults aged ≥35 y in outpatient clinics. Nonetheless, we simulated adding the cost of systematic hypertension screening of adults aged 35–84 y in outpatient clinics: twice yearly for adults without diabetes or chronic kidney disease and systolic BP of 130–139 mm Hg or diastolic BP of 85–89 mm Hg and annually for those below 130 mm Hg systolic and 85 mm Hg diastolic. The expanded screening program was simulated by adding screening visit direct costs (Table 1) [20] for the following groups: (1) people unaware of their hypertension diagnosis aged 35–84 y in 2015, (2) ongoing screening of undiagnosed persons (twice yearly if prior screening result was 130–139/85–89 mm Hg and annually if <130/85 mm Hg), and (3) waves of 35-y-olds in the years 2015–2025. The main cost of implementing China’s zero-profit essential medicines program would be replacing physician’s income derived in the past from adding a personal service fee to every prescription dispensed. Based on policies proposed for Chongching and Tianjin provinces, we assumed that the government would cover the cost of returning 15% of pharmaceutical expenditures as payments to physicians prescribing according to the essential medicines program rules [29]. Lastly, following the findings of a World Health Organization CHOosing Interventions that are Cost Effective (WHO-CHOICE) analysis of program costs, we assumed that a clinic-based prevention program would require an additional 5% of total intervention costs for program administration [30].

Analyses were interpreted from a payer’s perspective. Effectiveness (measured as QALYs gained, reductions in coronary heart disease and stroke events), screening, treatment, monitoring, and total costs (inclusive of acute and chronic CVD treatment costs saved) were simulated over the years 2015–2025 and averaged to annual estimates. All future costs and QALYs were discounted at 3% annually. Incremental cost-effectiveness ratios (ICERs) were calculated by dividing the incremental change in CVD costs by incremental change in QALYs. All results reported as cost saving describe less costly and more effective strategies. Results are reported in 2015 international dollars and 2015 Chinese renminbi (RMB) according to the exchange rate published by the World Bank, based on PPP methods (1.00 yuan = Int$0.28; Int$1.00 = 3.52 yuan). The ICER threshold for cost-effectiveness was based on the WHO-CHOICE-recommended gross domestic product (GDP) per capita-indexed cost-effectiveness threshold of highly cost effective (< 1 x GDP per capita). A cost-effectiveness acceptability curve also assessed the probability of cost-effectiveness over a range of willingness to pay thresholds, including a threshold of 2 x GDP per capita. Basing the conversion rate on PPP, GDP per capita for China was assumed to be Int$11,900 (38,450 RMB)].

### Exploratory and Sensitivity Analyses

One-way sensitivity analyses examined higher and lower medication adherence rates, ranges of uncertainty surrounding the relative risk of coronary heart disease and stroke per mm Hg lower BP, the BP lowering effect of antihypertensive medications (mm Hg change), medication costs, and the probability of side effects (Table 1). Three alternate medication cost assumptions were explored: (1) the average of lowest national essential medicines list prices per drug class, (2) median prices from the Shanghai municipality essential medicines list (highest cost assumption), and (3) median prices from the Yunnan province essential medicines list (lowest cost assumption, S1 Text). For stage two hypertension patients without diabetes, mean drug costs ranged from Int$44–Int$46 (Yunnan, 281–295 RMB) to Int$193–Int$236 (Shanghai, 1,227–1,505 RMB); stage one mean drug costs ranged from Int$ 19–Int$22 (Yunnan, 122–137 RMB) to Int$46–Int$92 (Shanghai, 296–589 RMB). An exploratory analysis repeated the main simulations after recalibrating stroke and coronary heart disease incidence to match higher cause-specific mortality targets for stroke and coronary heart disease reported by the China Ministry of Health (S1 Text). Lacking specific data on indirect costs to patients, we tested the sensitivity of the results to possible indirect costs by adding 10% higher cost for treatment and monitoring and adding 50% higher cost to acute CVD event costs. Lastly, we assessed the cost-effectiveness of hypertension treatment, including medication, monitoring, and side effect costs but excluding screening, program administration, and implementation costs.

In a probabilistic (Monte Carlo) analysis, 1,000 random draws were taken of the uncertainty distributions for systolic BP relative risk, BP lowering with treatment, quality of life penalties, total treatment costs (inclusive of medications, monitoring, and side effect costs), case fatality, background CVD treatment costs, population mean BP, hypertension prevalence, and antihypertensive drug use (S1 Text and S6 Table). The uncertainty intervals reported do not include the following sources of uncertainty: variation in program administration costs, Essential Medicines implementation costs, screening costs, or CVD incidence or prevalence, because uncertainty distribution estimates were not available for these inputs. Multivariable probabilistic analyses resulted in 1,000 cost and QALY pairings that were used to calculate 95% uncertainty intervals for incremental costs, QALYs, ICER estimates and the proportion of ICERs that were cost saving or cost-effective, and probability of cost-effectiveness at different willingness-to-pay thresholds.

In order to quantify lifetime benefits of hypertension treatment, we simulated a cohort of 20 million 35–44-y-old adults until death or reaching the age of 84 y. By comparing with a status quo cohort simulation, we tabulated lifetime gains in discounted life years and QALYs gained and costs incurred per person-year of hypertension treatment initiated in untreated eligible adults at whatever age they first met diagnostic criteria. National program costs were not included in the cohort simulation.

## Results

If the Chinese government systematically screened adults aged 35–84 years for hypertension, it would require an investment of about Int$962 million (3.4 billion RMB) in 2015 to screen adults unaware of an existing hypertension diagnosis, and about Int$65 billion U.S. annually (231 billion RMB) to screen adults currently without hypertension and all persons becoming 35 years of age after 2015 for incident cases of hypertension during 2015-2025. Assuming use of low-cost medications from the national essential medicines list, treating hypertensive adults with a prior diagnosis of CVD for secondary prevention was projected to prevent about 111,000 cardiovascular events yearly over 2015–2025. Treating all previously untreated adults with stage two hypertension for primary prevention, along with secondary prevention treatment in CVD patients, was projected to avert about 583,000 strokes and 93,000 myocardial infarctions and gain about 934,000 QALYs annually compared with the status quo (Table 2). Treating all hypertension (stage one and stage two; primary and secondary prevention) would prevent about 803,000 CVD events and gain about 1.2 million QALYs annually compared with the status quo.

**Table 2:** Effectiveness and cost-effectiveness of implementing different BP control guidelines in untreated Chinese adults aged 35–84 y with hypertension, averaged from the projections for 2015–2025, the CVD Policy Model-China. Each successive strategy is compared with the prior strategy. Results are in 2015 international dollars and 2015 Chinese RMB. All results reported as cost-saving describe strategies projected to be less costly and more effective than the prior strategy. Ninety-five percent uncertainty intervals were calculated from the results of 1,000 probabilistic simulations.

Strategy*	Annual Number of Hypertensive Adults Newly Treated	Annual Total Stroke Events (95% Uncertainty Interval)	Annual Total Myocardial Infarction Events (95% Uncertainty Interval)	Annual QALYs, Millions (95% Uncertainty Interval)	Annual CVD Costs, Millions (95% Uncertainty Interval) †	ICERs (95% Uncertainty Interval) †
**Status Quo case** (projected China 2015–2025)	Not applicable	5,548,000	1,511,000	653.92	Int$74,200	Not applicable
					¥261,300	
**Control BP in all persons living with CHD or stroke** (base case)	5,807,000	5,458,000 (5,394,000–5,500,000)	1,490,000 (1,478,000–1,498,000)	654.00 (653.76–654.10)	Int$74,000 (Int$73,500–Int$74,400)	Cost-saving (cost saving—cost saving)
					¥260,300 (¥258,600–¥261,800)	Cost-saving (cost saving—cost saving)
**Strategy 1:** Treat all stage two hypertension patients to goal of <140/90 if age 35–64 y, goal of 150/90 if age ≥65	62,258,000	4,965,000 (4,789,000–5,124,000)	1,417,000 (1,393,000–1,435,000)	654.85 (654.50–655.07)	Int$81,700 (Int$80,500–Int$82,900)	Int$9,000 (Int$7,000–Int$12,000)
					¥287,700 (¥283,200–¥291,800)	¥32,000 (¥24,000–¥ 42,000)
**Strategy 2:** Treat all stage two and stage one, goal of <140/90 for ages 35–64 y, goal of 150/90 if age ≥65	173,950,000	4,858,000 (4,644,000–5,035,000)	1,398,000 (1,368,000–1,419,000)	655.10 (654.72–655.35)	Int$85,000 (Int$83,400–Int$86,500)	Int$13,000§ (Int$10,000–Int$18,000)
					¥299,300 (¥293,700–¥304,400)	¥47,000 (¥34,000–¥64,000)

* All guideline strategies affect adults not previously treated for hypertension only, i.e., “aware/treated/uncontrolled” population not treated.

^†^ To convert cost input to Chinese currency, multiply by PPP rate (in this case, 3.52). To convert to $US using the current official exchange rate, multiply by (PPP/exchange rate), for example, 3.52/6.20, or by 5.68.

^§^ Less than 2 x China’s GDP per capita.

**Fig 2:**
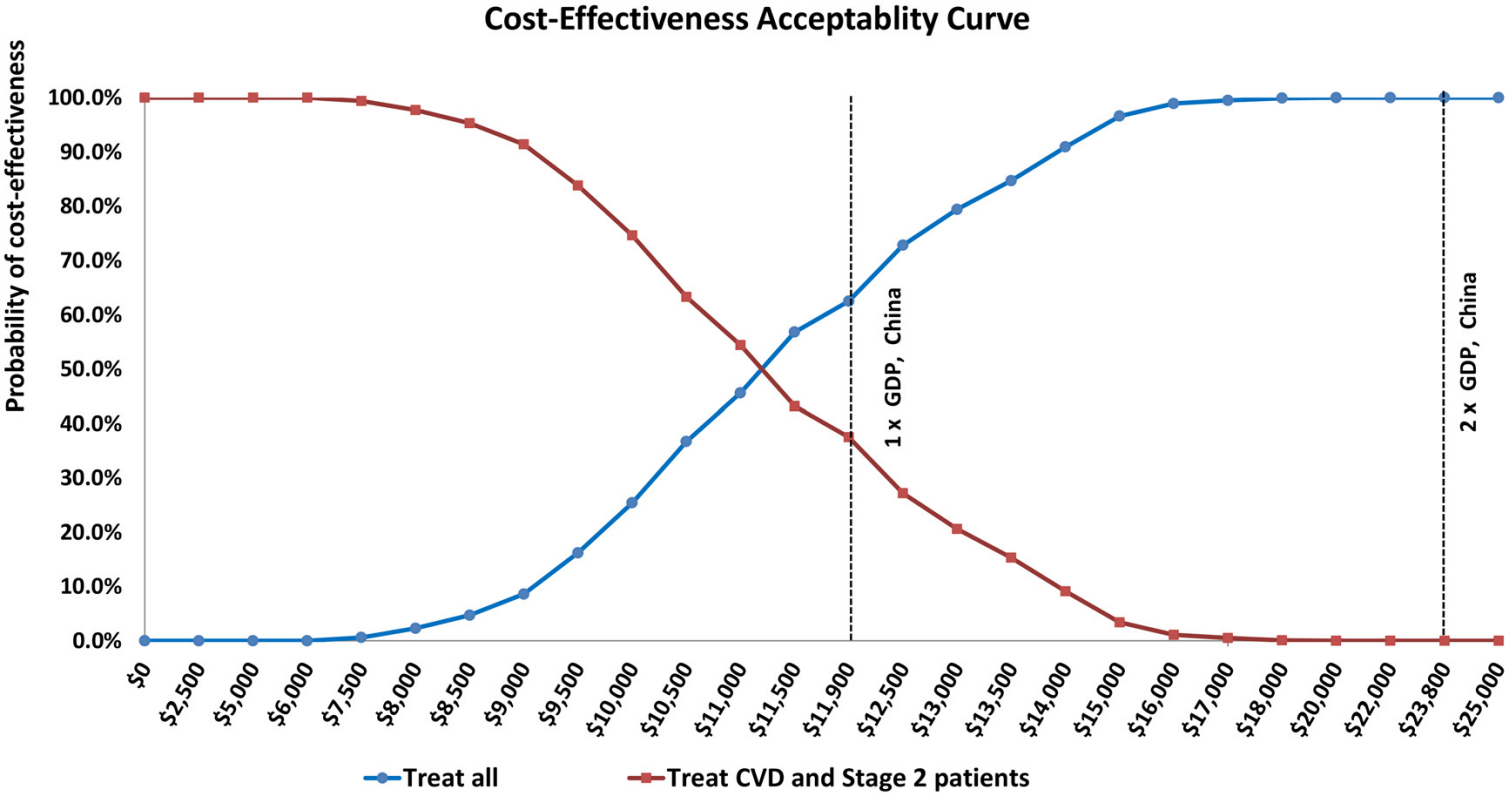
Cost-effectiveness acceptability curves comparing treating all untreated hypertensive adults (blue) with treating only untreated CVD patients and adults with stage 2 hypertension but without CVD (red). The threshold for cost-effective in China assumed for this analysis is labeled at Int$11,900 (China’s GDP per capita; conversion to US dollars from Chinese RMB based on PPP). Twice China’s GDP is also labelled at Int$23,800.

Treating hypertension in CVD patients was projected to save costs in 100% of probabilistic simulations. Incrementally adding treatment of stage two patients was projected to be cost-effective (Int$9,000 per QALY gained, [95% uncertainty range Int$7,000 to Int$12,000], Table 2 and S7 Table). Treating all untreated hypertensive patients (primary and secondary prevention) was projected to be borderline cost-effective compared with treating stage two and CVD patients alone (about Int$13,000 per QALY gained, [Int$10,000 to Int$18,000]). At a willingness-to-pay threshold of the GDP per capita of China (Int$11,900 in 2015), treating all hypertensives was the most cost-effective strategy in 63% of probabilistic simulations (Fig 2). At thresholds of Int$19,000 and above, treating all hypertensive adults was the most cost-effective strategy in 100% of simulations.

Of all one-way sensitivity analyses, assuming 25% medication adherence by patients, high Shanghai drug costs, or low medication efficacy led to the most unfavorable cost-effectiveness results (treating all hypertension about Int$47,000, Int$37,000, and Int$27,000 per QALY gained, respectively, Table 3). Assuming lower medication costs, lower monitoring costs (Int$1 less per visit), or achieving the same health gains using less frequent screening (two fewer visits for stage two, one fewer visit for stage one) led to exceptionally low-cost projections. Adding 10% higher treatment and monitoring costs or 50% acute CVD event costs to reflect possible indirect costs to patients did not change the results substantially. When screening, program administration, and implementation costs were excluded, adding primary prevention treatment of stage two hypertension was cost saving, and treating all hypertensive adults remained cost-effective (Int$12,000 per QALY gained).

**Table 3:** One-way sensitivity analysis of hypertension treatment inputs. All estimates are ICERs, compared with the prior strategy. Results are in 2015 international dollars (2015 Chinese RMB). All results reported as cost saving describe strategies projected to be less costly and more effective than the prior strategy.

Strategy	Strategy 1: Treat all stage two hypertension patients to goal of <140/90 if age 35–64 y, goal of 150/90 if age ≥65, in addition to CVD patients	Strategy 2: Treat stage two and stage one, goal <140/90 if age 35–64 y, goal of 150/90 if age ≥65, in addition to CVD patients
Comparator for ICER	Treat only CVD patients (base case)	Strategy 1
Main assumptions simulations	Int$9,000 (¥32,000)Δ	Int$13,000 (¥47,000)§
Assume higher CVD incidence	Int$7,000 (¥26,000)Δ	Int$10,000 (¥37,000)Δ
Sex		
Males	Int$7,000 (¥24,000)Δ	Int$12,000 (¥41,000)Δ
Females	Int$12,000 (¥44,000)Δ	Int$15,000 (¥54,000)§
Relative risk with change in BP		
Lower 95% confidence interval of RRs	Int$17,000 (¥61,000)Δ	Int$15,000 (¥53,000)Δ
Upper 95% confidence interval of RRs	Int$5,000 (¥18,000)Δ	Int$12,000 (¥42,000)§
Range in efficacy of antihypertensive agents (change in BP with treatment)		
Upper	Int$7,000 (¥23,000)Δ	Int$8,000 (¥29,000)Δ
Lower	Int$14,000 (¥49,000)Δ	Int$27,900 (¥96,000)**
Adherence to any pharmaceutical therapy		
75%	Int$7,000 (¥25,000)Δ	Int$14,000 (¥49,000)§
25%	Int$25,000 (¥87,000)**	Int$47,000 (¥165,000)¶
Range of severity of side effects of antihypertensive medications		
Upper	Int$9,000 (¥32,000)Δ	Int$14,000 (¥ 48,000)§
Lower	Int$600 (¥2,000)Δ	Int$13,000 (¥ 46,000)Δ
Range of drug costs		
Low cost: Average of lowest national essential medicines costs per antihypertensive class	Int$8,000 (¥27,000)Δ	Int$10,000 (¥131,000)Δ
Lowest cost: Average of median costs per antihypertensive class, Yunnan province essential medicines list	Int$9,000 (¥31,000)Δ	Int$12,000 (¥34,000)Δ
High cost: Average of median costs per antihypertensive class, Shanghai municipality essential medicines list	Int$19,000 (¥67,000)§	Int$37,000 (¥131,000)¶
Range of monitoring costs		
Lower monitoring cost*	Int$8,000 (¥31,000)Δ	Int$12,000 (¥42,000)Δ
Less frequent monitoring†	Int$8,000 (¥29,000)Δ	Int$10,000 (¥35,000)Δ
Higher monitoring cost	Int$9,000 (¥32,000)Δ	Int$14,000 (¥48,000)§
More frequent monitoring‡	Int$10,000 (¥34,000)Δ	Int$21,000 (¥75,000)§
Hypothetical cost scenarios		
Increase hypertension treatment costs 10%	Int$10,000 (¥34,000)Δ	Int$15,000 (¥53,000)§
Increase CVD treatment costs 50%	Int$7,000 (¥24,000)Δ	Int$11,000 (¥40,000)Δ
Increase both cost inputs above	Int$7,000 (¥26,000)Δ	Int$13,000 (¥46,000)Δ
Without costs of screening, program administration, or implementation (medication, monitoring, and side effect costs only)	Cost-saving	Int$12,000 (¥42,000)Δ

* WHO CHOICE lowest outpatient visit cost for China

^†^ Stage two twice yearly, stage one once yearly

^‡^ Stage two four times yearly, stage one three times yearly

^Δ^ Less than 1 x China’s 2015 GDP per capita (<Int$11,900; international dollars)

^§^ Less than 2 x China’s 2015 GDP per capita and greater than 1 x GDP per capita (≥Int$11,906 and < Int$23,812)

**Less than 3 x China’s GDP per capita and greater than 2 x GDP per capita (≥Int$23,812 and <Int$35,718)

^¶^ Greater than 3 x China’s GDP per capita (≥Int$35,718)

For the cohort simulation starting at ages 35–44 y, hypertension treatment starting at the age at meeting diagnostic criteria led to an average lifetime gain of about five healthy days of life (4.8 quality-adjusted d and 4.2 total d) and an average lifetime cost of Int$1.80 (6.34 RMB).

## Discussion

In this analysis of hypertension control for CVD prevention in China, we found that controlling hypertension in adults aged 35–84 y could prevent about 800,000 cardiovascular events annually and be borderline cost-effective. Strengthening one pillar of China’s 2009 health reform—affordable essential antihypertensive medications—appeared to be crucial for achieving population-wide hypertension control at low cost.

Very few past studies have estimated the cost-effectiveness of hypertension treatment in China, and to our knowledge, ours is the first to assess cost-effectiveness by balancing program and intervention costs with projected downstream benefits of prevented CVD events. In a mathematical modeling study, Lim et al. estimated that scaling up a multidrug CVD prevention program, including aspirin and a statin along with an antihypertensive medication, would cost about US$55 per patient treated and would be relatively expensive, costing China about one US dollar per capita population in 2006 [27]. That analysis did not report on hypertension treatment specifically and likely overestimated the net cost of the program by not including cost saved by preventing or delaying CVD events. Three cost-effectiveness analyses conducted in China used primary data to demonstrate that implementing community health center-based hypertension management programs is effective and inexpensive, but cost-effectiveness was not measured based on prevented CVD or life-year gains [31–33].

The methods and reporting of this study conform to Consolidated Health Economic Evaluation Reporting Standards (CHEERS) [34] and Quality of Health Economic Studies Instrument standards recommended for cost-effectiveness analyses of CVD risk-factor guidelines [35]. China-specific demographic, epidemiologic, and health care cost data were used whenever possible. Effectiveness assumptions were grounded in a large meta-analysis of randomized antihypertensive medication treatment trials. However, all computer simulation studies are limited by reliance on numerous assumptions derived from diverse study designs and samples. Some model inputs were derived from studies of non-Chinese CVD patients and may not represent the general Chinese population. This analysis was limited in that educational and dietary measures for lowering BP were not compared with pharmacologic treatment, nor was hypertension treatment assessed in combination with treatment of elevated serum cholesterol levels [27,36]. We varied drug costs according to published essential medicines lists using median prices within antihypertensive drug classes, so our medication cost inputs do not reflect the frequency with which specifically priced agents are actually prescribed. We did not model the possibility that the practice of charging additional costs to patients by individual prescribers will persist even as the essential medicines program is implemented. We did not account for all of the costs of scaling up a hypertension treatment program (including training and infrastructure costs), specific out-of-pocket and indirect costs that would be incurred by patients participating in hypertension treatment, or indirect costs avoided as a result of prevented CVD.

While China rapidly expanded health insurance coverage nationally within the past decade, many Chinese adults still have limited access to hypertension screening and follow-up for hypertension treatment and monitoring. For example, in the New Rural Cooperative Medical Scheme, which now covers over 95% of the rural population, most coverage is for inpatient hospitalizations, and the costs of basic medical services, including hypertension education, screening, treatment, and monitoring, are not usually covered [4,37]. The results of our analysis suggest that expanding the scope of hypertension treatment would be borderline cost-effective for a government payer (around China’s per capita GDP) even if the costs of systematic screening of adults ages 35–84-y-old and essential medicines program implementation costs were added to medication and monitoring costs.

Our results were most sensitive to assumptions about medication costs and patient adherence to medications, both of which are influenced by drug pricing. It is estimated that of the 5% of China’s GDP that is spent on health care, 42% is spent on pharmaceuticals, a much higher proportion than is spent in high-income nations (15% on average) [7]. Because medication costs are usually paid out-of-pocket by patients with hypertension, local and national governments do not directly feel the impact of high drug costs [5]. However, high drug costs likely have a big impact at the level of individual households and therefore indirectly on the national economy [19]. Additionally, Chinese patients are reluctant to pay out-of-pocket for antihypertensive medications [5], and studies of Chinese patients have shown that out-of-pocket drug costs reduce medication adherence among patients with hypertension [3] and CVD [38]. Therefore, the Chinese government should work to ensure that the expense of scaling up a national hypertension control program is not borne in large part by patients. A subsidized antihypertensive medications program in Shandong province improved medication adherence dramatically [3] and might be successfully scaled up to a national policy.

Our computer simulation modeling study projected that treating hypertension in untreated Chinese adults could prevent about 800,000 CVD events annually and was cost-effective in over 63% of simulations. Cost-effectiveness was particularly sensitive to medication adherence and antihypertensive drug costs, implying that full implementation of the essential medicines program and subsidized drug costs programs will be important for reaping the benefits of improved hypertension control in China.

## Supporting Information

S1 CHEERS Checklist Standard checklist for cost-effectiveness studies. (DOCX)

S1 Fig Scatterplot of probabilistic sensitivity analysis results. (TIF)

S1 Table Results of the systolic BP calibration exercise. (DOCX)

S2 Table Model calibration results. (DOCX)

S3 Table Model validation results. (DOCX)

S4 Table Sequential changes in BP with successive standard-dose medications. (DOCX)

S5 Table Detailed effectiveness and drug cost assumptions for systolic BP. (DOCX)

S6 Table Distributions of main input parameters used in probabilistic sensitivity analyses. (DOCX)

S7 Table Cost effectiveness hypertension control in Chinese adults with and without adding separate components of program costs. (DOCX)

S1 Text Supplementary appendix. (DOCX)

S1 User Agreement Collaboration agreement used by CVD Policy Model software users. (DOC)

## Abbreviations

BMI: body mass index
BP: blood pressure
CHEERS: Consolidated Health Economic Evaluation Reporting Standards
CMCS: China Multi-provincial Cohort Study
CVD: cardiovascular disease
GDP: gross domestic product
HDL: high-density lipoprotein
ICER: incremental cost-effectiveness ratio
IDH: isolated diastolic hypertension
InterASIA: International Collaborative Study of Cardiovascular disease in Asia
LDL: low-density lipoprotein
NHS: National Health Service
PPP: purchasing power parity
PURE: Prospective Urban Rural Epidemiology
QALY: quality-adjusted life year
RMB: renminbi
SHEP: Systolic Hypertension in the Elderly Program
WHO CHOICE: World Health Organization CHOosing Interventions that are Cost Effective
