# Supplementary material for: The Cost-Effectiveness of Low-Cost Essential Antihypertensive Medicines for Hypertension Control in China: A Modelling Study
Source: PLoS Med. 2015 Aug 4;12(8):e1001860. doi: 10.1371/journal.pmed.1001860 (PMC4524696; doi:10.1371/journal.pmed.1001860)
Supplement: S1 CHEERS Checklist — (DOCX) [file pmed.1001860.s001.docx]

**CHEERS Checklist**

**Items to include when reporting economic evaluations of health interventions**

The **ISPOR CHEERS Task Force Report**, *Consolidated Health Economic Evaluation Reporting Standards (CHEERS)—Explanation and Elaboration: A Report of the ISPOR Health Economic Evaluations Publication Guidelines Good Reporting Practices Task Force,* provides examples and further discussion of the 24-item CHEERS Checklist and the CHEERS Statement. It may be accessed via the *Value in Health* or via the ISPOR Health Economic Evaluation Publication Guidelines – CHEERS: Good Reporting Practices

webpage: <http://www.ispor.org/TaskForces/EconomicPubGuidelines.asp>

**Section/item Item**

**No**

**Recommendation Reported**

**on page No/**

**line No**

**Title and abstract**

Title 1 Identify the study as an economic evaluation or use more specific terms such as “cost-effectiveness analysis”, and describe the interventions compared.

Title page

Abstract 2 Provide a structured summary of objectives, perspective, setting, methods (including study design and inputs), results (including base case and uncertainty analyses), and

Abstract pages 3,4

conclusions.

**Introduction** Background and objectives

**Methods**

Target population and subgroups

3 Provide an explicit statement of the broader context for the study.

Page 5

Present the study question and its relevance for health policy or practice decisions.

Pages 6-13

4 Describe characteristics of the base case population and

subgroups analysed, including why they were chosen.

Setting and location 5 State relevant aspects of the system(s) in which the decision(s)

Page 6

need(s) to be made.

Study perspective 6 Describe the perspective of the study and relate this to the costs being evaluated.

Page 11

Comparators 7 Describe the interventions or strategies being compared and state why they were chosen.

Page 8

Time horizon 8 State the time horizon(s) over which costs and consequences are being evaluated and say why appropriate.

Pages 8, 13

Discount rate 9 Report the choice of discount rate(s) used for costs and

Table 1, page 11

outcomes and say why appropriate.

Choice of health outcomes

Measurement of effectiveness

10 Describe what outcomes were used as the measure(s) of benefit in the evaluation and their relevance for the type of analysis performed.

Page 6

11a *Single study-based estimates:* Describe fully the design features of the single effectiveness study and why the single

Not applicable

study was a sufficient source of clinical effectiveness data.

Measurement and valuation of preference based outcomes Estimating resources and costs

Currency, price date, and conversion

11b *Synthesis-based estimates:* Describe fully the methods used for identification of included studies and synthesis of clinical effectiveness data.

Pages 7-8

12 If applicable, describe the population and methods used to elicit preferences for outcomes.

Not applicable

13a *Single study-based economic evaluation:* Describe approaches used to estimate resource use associated with the alternative interventions. Describe primary or secondary research methods for valuing each resource item in terms of its unit cost.

Not applicable

Describe any adjustments made to approximate to opportunity costs.

13b *Model-based economic evaluation:* Describe approaches and data sources used to estimate resource use associated with model health states. Describe primary or secondary research methods for valuing each resource item in terms of its unit cost. Describe any adjustments made to approximate to opportunity costs.

Pages 9-11 (note: indirect and opportunity costs not considered)

14 Report the dates of the estimated resource quantities and unit costs. Describe methods for adjusting estimated unit costs to the year of reported costs if necessary. Describe methods for converting costs into a common currency base and the

Table 1 (pages 10-11)

exchange rate.

Choice of model 15 Describe and give reasons for the specific type of decision- analytical model used. Providing a figure to show model structure is strongly recommended.

Page 6-7, Figure 1

Assumptions 16 Describe all structural or other assumptions underpinning the decision-analytical model.

Figure 1, pages 6-7, appendix

Analytical methods 17 Describe all analytical methods supporting the evaluation. This could include methods for dealing with skewed, missing, or censored data; extrapolation methods; methods for pooling data; approaches to validate or make adjustments (such as half cycle corrections) to a model; and methods for handling

Pages 11-13

population heterogeneity and uncertainty.

**Results**

Study parameters 18 Report the values, ranges, references, and, if used, probability distributions for all parameters. Report reasons or sources for distributions used to represent uncertainty where appropriate. Providing a table to show the input values is strongly

Table 1

recommended.

Incremental costs and outcomes

Characterising uncertainty

19 For each intervention, report mean values for the main categories of estimated costs and outcomes of interest, as well as mean differences between the comparator groups. If

Table 1, Table 2, Figure 2

applicable, report incremental cost-effectiveness ratios.

20a *Single study-based economic evaluation:* Describe the effects of sampling uncertainty for the estimated incremental cost and

Table 2, Figure 2

incremental effectiveness parameters, together with the impact

Characterising heterogeneity

**Discussion** Study findings, limitations, generalisability, and current knowledge

**Other**

of methodological assumptions (such as discount rate, study perspective).

20b *Model-based economic evaluation:* Describe the effects on the results of uncertainty for all input parameters, and uncertainty

Pages 13-14, Table 2, Figure 2

related to the structure of the model and assumptions.

21 If applicable, report differences in costs, outcomes, or cost- effectiveness that can be explained by variations between subgroups of patients with different baseline characteristics or other observed variability in effects that are not reducible by more information.

Table 3

22 Summarise key study findings and describe how they support the conclusions reached. Discuss limitations and the generalisability of the findings and how the findings fit with current knowledge.

Pages 15-18

Source of funding 23 Describe how the study was funded and the role of the funder in the identification, design, conduct, and reporting of the

Page 19

analysis. Describe other non-monetary sources of support. Conflicts of interest 24 Describe any potential for conflict of interest of study

Page 19

contributors in accordance with journal policy. In the absence

of a journal policy, we recommend authors comply with

International Committee of Medical Journal Editors

recommendations.

For consistency, the CHEERS Statement checklist format is based on the format of the CONSORT

statement checklist

The **ISPOR CHEERS Task Force Report** provides examples and further discussion of the 24-item CHEERS Checklist and the CHEERS Statement. It may be accessed via the *Value in Health* link or via the ISPOR Health Economic Evaluation Publication Guidelines – CHEERS: Good Reporting Practices

webpage: <http://www.ispor.org/TaskForces/EconomicPubGuidelines.asp>

The citation for the CHEERS Task Force Report is:

Husereau D, Drummond M, Petrou S, et al. Consolidated health economic evaluation reporting standards (CHEERS)—Explanation and elaboration: A report of the ISPOR health economic evaluations publication guidelines good reporting practices task force. Value Health 2013;16:231-50.
