# Supplementary figures and images for: The Cost-Effectiveness of Low-Cost Essential Antihypertensive Medicines for Hypertension Control in China: A Modelling Study
Source: PLoS Med. 2015 Aug 4;12(8):e1001860. doi: 10.1371/journal.pmed.1001860 (PMC4524696; doi:10.1371/journal.pmed.1001860)

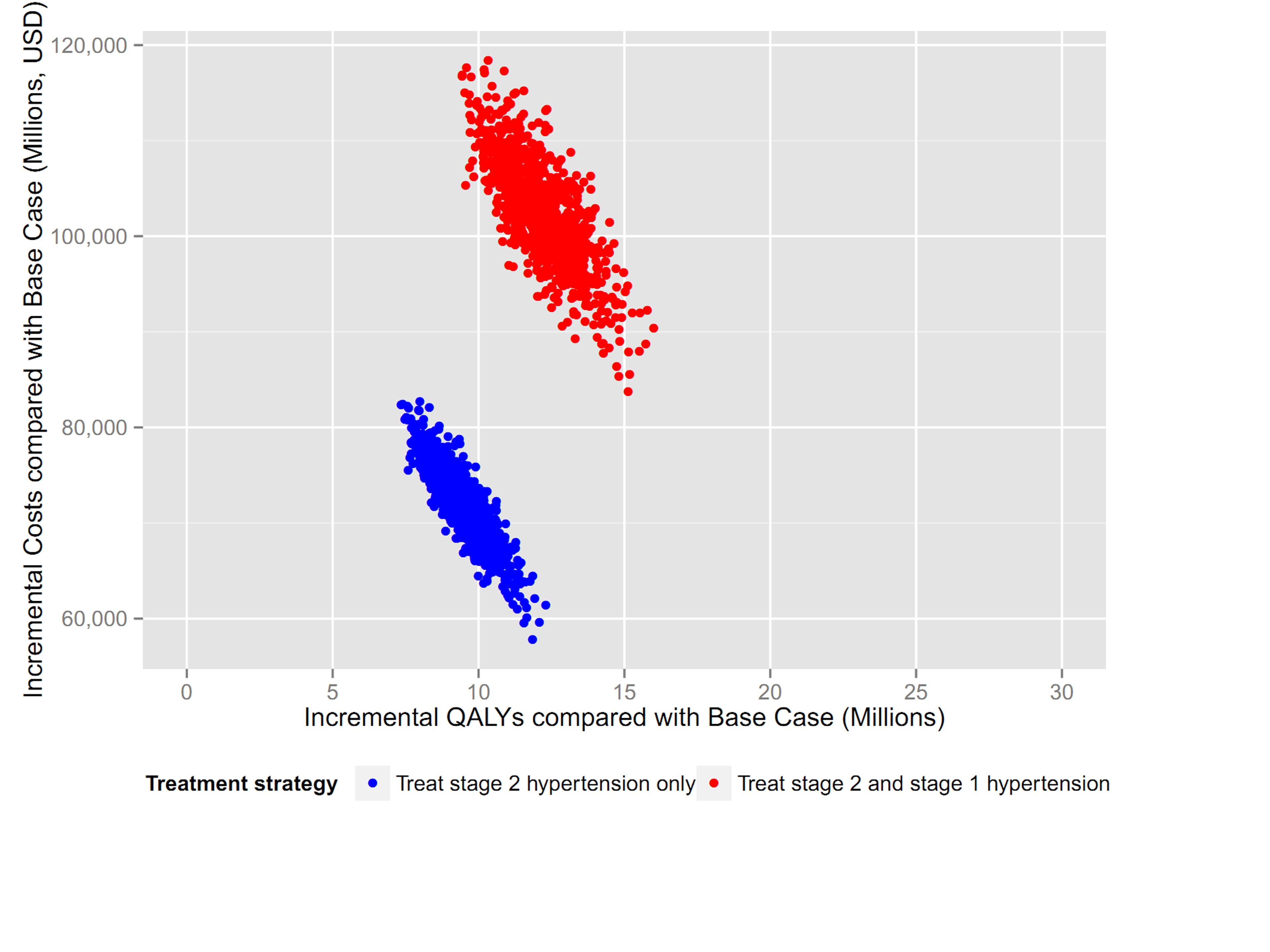

Supplement: S1 Fig — (TIF) [file pmed.1001860.s002.tif]
