# Supplementary material for: The Cost-Effectiveness of Low-Cost Essential Antihypertensive Medicines for Hypertension Control in China: A Modelling Study
Source: PLoS Med. 2015 Aug 4;12(8):e1001860. doi: 10.1371/journal.pmed.1001860 (PMC4524696; doi:10.1371/journal.pmed.1001860)
Supplement: S1 Table — (DOCX) [file pmed.1001860.s003.docx]

**S1 Table 1. Results of the systolic blood pressure calibration exercise. CMCS = China Multi-provincial Cohort Study. PSC = Prospective Studies Collaboration. CVDPM = CVD Policy Model inputs and simulation outputs. NA = not available from data source. Due to limited data availability for ages 85-94 years, the age 75-84 estimate was used for that group.**

|  | | Beta coefficients (per 1.0 mm Hg systolic blood pressure or 0.5 mm Hg diastolic blood pressure) | | | | | | Relative risks (after systolic blood pressure change of 10 mm Hg or diastolic blood pressure change of 5 mm Hg) | | | | | |
| --- | --- | --- | --- | --- | --- | --- | --- | --- | --- | --- | --- | --- | --- |
|  | | CHD | | | Stroke | | | CHD | | | Stroke | | |
| Age/sex category | Mean change in SBP (mm Hg) | CMCS  default | PCS target | CVDPM  (input) | CMCS  default | PCS target | CVDPM  (input) | CMCS  default | PCS target | CVDPM (output) | CMCS  default | PCS target | CVDPM (output) |
| Males |  |  |  |  |  |  |  |  |  |  |  |  |  |
| 35-44 | 9.57 | .0150 | .0361 | .0325 | .0310 | .0513 | .0500 | .86 | .71 | .73 | .73 | .61 | .60 |
| 45-54 | 10.38 | .0150 | .0353 | .0310 | .0310 | .0496 | .0460 | .86 | .70` | .72 | .73 | .61 | .59 |
| 55-64 | 10.38 | .0150 | .0330 | .0300 | .0310 | .0453 | .0420 | .86 | .71 | .73 | .73 | .63 | .62 |
| 65-74 | 10.88 | .0150 | .0285 | .0265 | .0310 | .0385 | .0370 | .86 | .74 | .76 | .73 | .66 | .66 |
| 75-84 | 11.58 | NA | .0228 | .0230 | NA | .0273 | .0267 | NA | .77 | .77 | NA | .74 | .73 |
| Females |  |  |  |  |  |  |  |  |  |  |  |  |  |
| 35-44 | 9.87 | 0.110 | .0360 | .0320 | .0260 | .0513 | .0470 | .90 | .70 | .72 | .77 | .60 | .60 |
| 45-54 | 10.35 | 0.110 | .0353 | .0320 | .0260 | .0496 | .0450 | .90 | .70 | .72 | .77 | .61 | .60 |
| 55-64 | 10.94 | 0.110 | .0330 | .0295 | .0260 | .0452 | .0414 | .90 | .70 | .72 | .77 | .62 | .61 |
| 65-74 | 11.68 | 0.110 | .0285 | .0245 | .0260 | .0385 | .0345 | .90 | .73 | .75 | .77 | .65 | .65 |
| 75-84 | 12.67 | NA | .0228 | .0228 | NA | .0269 | .0267 | NA | .76 | .75 | NA | .72 | .71 |
