# Supplementary material for: The Cost-Effectiveness of Low-Cost Essential Antihypertensive Medicines for Hypertension Control in China: A Modelling Study
Source: PLoS Med. 2015 Aug 4;12(8):e1001860. doi: 10.1371/journal.pmed.1001860 (PMC4524696; doi:10.1371/journal.pmed.1001860)
Supplement: S2 Table — (DOCX) [file pmed.1001860.s004.docx]

**S1Table 2. Comparison of CVD Policy Model trial simulation to Law Morris Wald meta-analysis of anti-hypertensive medication treatment trials, both assuming a systolic blood pressure change of 10 mm Hg or a diastolic blood pressure change of 5 mm Hg.**

| Outcome | Law, Morris, and Wald meta-analysis estimate | CVD Policy Model main estimate  Males | CVD Policy Model main estimate  Females |
| --- | --- | --- | --- |
| Coronary heart disease | 0.78 (0.73—0.83) | Ages 35-74 years: 0.75  Ages 35-64 years: 0.74 | Ages 35-74 years: 0.74  Ages 35-64 years: 0.73 |
| Stroke | 0.59 (0.52—0.67) | Ages 35-74 years: 0.64  Ages 35-64 years: 0.62 | Ages 35-74 years: 0.63  Ages 35-64 years: 0.62 |
