# Supplementary material for: The Cost-Effectiveness of Low-Cost Essential Antihypertensive Medicines for Hypertension Control in China: A Modelling Study
Source: PLoS Med. 2015 Aug 4;12(8):e1001860. doi: 10.1371/journal.pmed.1001860 (PMC4524696; doi:10.1371/journal.pmed.1001860)
Supplement: S3 Table — (DOCX) [file pmed.1001860.s005.docx]

**S1Table 3. Results of a simulated trial of blood pressure treatment in participants in the Systolic Hypertension in the Elderly Program (SHEP) trial, using relative risk inputs and transition probabilities in the CVD Policy Model version used for the analysis.**

| **SHEP Population RR (SBP lowering of 12 mmHg for 5 years)** | | |
| --- | --- | --- |
| Outcome | Actual SHEP paper RR and 95% CI | CVD Policy Model five year simulation results with PSC calibrated SBP betas [overall rate ratio (95% uncertainty interval)*] |
| Incident stroke | 0.64 (0.50-0.82) | 0.70 (0.66-0.75) |
| Incident coronary heart disease† | 0.75 (0.60-0.94) | 0.76 (0.73-0.79) |

*95% uncertainty intervals derived from 1,000 probabilistic simulations that sampled from within the 95% confidence intervals of age- and sex-specific Prospective Cohorts Studies Collaboration beta coefficients.

†SHEP incident coronary heart disease defined as first-in-trial nonfatal acute myocardial infarction, coronary revascularization, or coronary heart disease death. CVD Policy Model incident coronary heart disease defined as first-ever nonfatal acute myocardial infarction, angina pectoris with or without coronary revascularization, or coronary heart disease death.
