# Supplementary material for: The Cost-Effectiveness of Low-Cost Essential Antihypertensive Medicines for Hypertension Control in China: A Modelling Study
Source: PLoS Med. 2015 Aug 4;12(8):e1001860. doi: 10.1371/journal.pmed.1001860 (PMC4524696; doi:10.1371/journal.pmed.1001860)
Supplement: S4 Table — (DOCX) [file pmed.1001860.s006.docx]

**S1 Table 4. Sequential changes in blood pressure with successive standard dose medications, based on the trials-based blood pressure change prediction formulas of Law, Morris, and Wald.** For one standard-dose medication, the formula for calculating the change in systolic blood pressure was [9.1+0.10×(BS-154)], and the formula for calculating the change in diastolic blood pressure was [5.1+0.11×(BD-97)], in which BS denotes the baseline systolic blood pressure and BD the baseline diastolic blood pressure. The formula for one half-standard dose was [6.7+0.078 x (BS-150)] for systolic and [3.7+0.088 x (BD-90)] for diastolic.

|  | Mean start BP | BP change 1 drug | new BP | BP change 2nd drug | new BP | BP change 3rd drug | new BP | BP change 4th drug | new BP | Final BP change | |
| --- | --- | --- | --- | --- | --- | --- | --- | --- | --- | --- | --- |
| **Systolic blood pressure (mm Hg changes)** |  |  |  |  |  |  |  |  |  | **< 60 years old** | **≥ 60 years old** |
| effect of 4 std dose | 185 | 12.2 | 172.8 | 11.0 | 161.8 | 9.9 | 151.9 | 8.9 | 143.0 | 42.0 | 33.1 |
| effect of 3.5 std doses | 175 | 11.2 | 163.8 | 10.1 | 153.7 | 9.1 | 144.6 | 6.3 | 138.4 | 36.6 | 21.3 |
| effect of 3 std doses | 165 | 10.2 | 154.8 | 9.2 | 145.6 | 8.3 | 137.4 |  |  | 27.6 | 19.4 |
| effect of 2 std doses | 155 | 9.2 | 145.8 | 8.3 | 137.5 |  |  |  |  | 17.5 |  |
| effect of 1 std dose | 155 | 9.2 | 145.8 |  |  |  |  |  |  | 9.2 |  |
| effect of 1 std dose | 147 | 8.4 | 138.6 |  |  |  |  |  |  | 8.4 |  |
| effect of 0.5 std dose | 142 | 6.1 | 135.9 |  |  |  |  |  |  | 6.1 |  |
| effect of 0.5 std dose | 155 | 7.1 | 147.9 |  |  |  |  |  |  |  | 7.1 |
| **Diastolic blood pressure (mm Hg changes)** |  |  |  |  |  |  |  |  |  | **All ages** | |
| effect of 3 std doses | 107 | 6.6 | 100.5 | 5.9 | 94.6 | 5.2 | 89.4 |  |  | 17.7 |  |
| effect of 3 std doses | 104 | 6.3 | 97.7 | 5.6 | 92.1 | 5.0 | 87.2 |  |  | 16.8 |  |
| effect of 3 std doses | 105 | 6.3 | 98.2 | 5.6 | 92.5 | 5.0 | 87.5 |  |  | 17.0 |  |
| effect of 3 std doses | 105 | 6.4 | 98.6 | 5.7 | 92.9 | 5.1 | 87.9 |  |  | 17.1 |  |
| effect of 3 std doses | 103 | 6.2 | 97.2 | 5.5 | 91.7 | 4.9 | 86.8 |  |  | 16.6 |  |
| effect of 3 std doses | 107 | 6.6 | 100.5 | 5.9 | 94.6 | 5.2 | 89.4 |  |  | 17.7 |  |
| effect one std dose | 94 | 5.2 | 89.0 |  |  |  |  |  |  | 5.2 |  |
| effect one std dose | 94 | 5.2 | 88.8 |  |  |  |  |  |  | 5.2 |  |
| effect one std dose | 93 | 5.1 | 88.0 |  |  |  |  |  |  | 5.1 |  |
| effect of 0.5 std dose | 94 | 4.1 | 90.1 |  |  |  |  |  |  | 4.1 |  |
| effect of 0.5 std dose | 94 | 4.1 | 89.9 |  |  |  |  |  |  | 4.1 |  |
