# Supplementary material for: The Cost-Effectiveness of Low-Cost Essential Antihypertensive Medicines for Hypertension Control in China: A Modelling Study
Source: PLoS Med. 2015 Aug 4;12(8):e1001860. doi: 10.1371/journal.pmed.1001860 (PMC4524696; doi:10.1371/journal.pmed.1001860)
Supplement: S5 Table — (DOCX) [file pmed.1001860.s007.docx]

**S1 Table 5. Detailed effectiveness and drug cost assumptions for systolic BP (age, sex, BP interval). Effects reported in this table reflect 75% adherence to medications.**

| **Men** |  | | | |  | | |  | | | |  | |  |  | | |  | | |
| --- | --- | --- | --- | --- | --- | --- | --- | --- | --- | --- | --- | --- | --- | --- | --- | --- | --- | --- | --- | --- |
|  | Stage 1 (140-159 mm Hg) | | | | | | | | | | |  | | Stage 2 ( ≥ 160 mm Hg) | | | | | | |
|  |  | | | | | |  | | |  | |  | |  | | |  | |  | |
| **Systolic BP interval** | 140-145 | | | | | | 146-148 | | | 149-159 | |  | | 160-169 | | | 170-179 | | >=180 | |
| **Number of standard dose drugs** | 0.5 | | | | | | 1.0 | | | 2.0 | |  | | 3.0 | | | 3.5 | | 4.0 | |
| **35-44 years** |  | | | | | |  | | |  | |  | |  | | |  | |  | |
| **percent of all untreated HT** | | | 41.1% | | | | 20.0% | | | 25.4% | |  | | 7.6% | | | 1.5% | | 4.4% | |
| **percent of stage** | | | 47.5% | | | | 23.1% | | | 29.4% | |  | | 56.2% | | | 11.0% | | 32.8% | |
| **BP change after treatment** | | | 4.2 | | | | 8.4 | | | 18.2 | |  | | 27.6 | | | 36.6 | | 42.0 | |
| **RR CHD after treatment** | | | 0.89 | | | | 0.79 | | | 0.61 | |  | | 0.47 | | | 0.37 | | 0.32 | |
| **RR stroke after treatment** | | | 0.86 | | | | 0.75 | | | 0.53 | |  | | 0.38 | | | 0.28 | | 0.23 | |
| **Annual anti-HT medication cost** | | | ¥ 33.68 | | | | ¥ 67.36 | | | ¥ 124.13 | |  | | ¥ 184.52 | | | ¥ 246.60 | | ¥ 269.43 | |
| **45-54 years** |  | | | | | |  | | |  | |  | |  | | |  | |  | |
| **percent of all untreated HT** | | | 35.3% | | | | 16.3% | | | 25.4% | |  | | 10.1% | | | 4.3% | | 8.6% | |
| **percent of stage** | | | 45.8% | | | | 21.2% | | | 33.0% | |  | | 44.0% | | | 18.8% | | 37.2% | |
| **BP change after treatment** | | | 4.2 | | | | 8.4 | | | 18.2 | |  | | 27.6 | | | 36.6 | | 42.0 | |
| **RR CHD after treatment** | | | 0.89 | | | | 0.80 | | | 0.61 | |  | | 0.47 | | | 0.37 | | 0.32 | |
| **RR stroke after treatment** | | | 0.86 | | | | 0.75 | | | 0.53 | |  | | 0.38 | | | 0.28 | | 0.23 | |
| **Annual anti-HT medication cost** | | | ¥ 33.68 | | | | ¥ 67.36 | | | ¥ 124.13 | |  | | ¥ 184.52 | | | ¥246.60 | | ¥ 269.43 | |
| **55-64 years** |  | | | | | |  | | |  | |  | |  | | |  | |  | |
| **percent of all untreated HT** | | | 20.6% | | | | 18.2% | | | 25.9% | |  | | 16.2% | | | 9.1% | | 9.9% | |
| **percent of stage** | | | 31.8% | | | | 28.2% | | | 40.0% | |  | | 45.9% | | | 25.9% | | 28.2% | |
| **BP change after treatment** | | | 4.2 | | | | 8.4 | | | 18.2 | |  | | 27.6 | | | 36.6 | | 42.0 | |
| **RR CHD after treatment** | | | 0.90 | | | | 0.81 | | | 0.63 | |  | | 0.49 | | | 0.39 | | 0.34 | |
| **RR stroke after treatment** | | | 0.88 | | | | 0.78 | | | 0.58 | |  | | 0.44 | | | 0.34 | | 0.29 | |
| **Annual anti-HT medication cost** | | | ¥ 33.68 | | | | ¥ 67.36 | | | ¥ 124.13 | |  | | ¥ 184.52 | | | ¥246.60 | | ¥ 269.43 | |
|  | | |  | | | |  | | |  | |  | |  | | |  | |  | |
| **65-74 years** |  | | | | | |  | | |  | |  | |  | | |  | |  | |
| **percent of all untreated HT** | | 23.0% | | | | | 9.4% | | | 29.0% | |  | | 14.6% | | | 7.7% | | 16.3% | |
| **percent of stage** | | 0.0% | | | | | 0.0% | | | 47.2% | |  | | 37.9% | | | 19.9% | | 42.1% | |
| **BP change after treatment** | | 0.0 | | | | | 0.0 | | | 8.4 | |  | | 18.2 | | | 27.6 | | 36.6 | |
| **RR CHD after treatment** | | 1.00 | | | | | 1.00 | | | 0.85 | |  | | 0.71 | | | 0.59 | | 0.50 | |
| **RR stroke after treatment** | | | | 1.00 | | | 1.00 | | | 0.81 | |  | | 0.63 | | | 0.50 | | | 0.40 |
| **Annual anti-HT medication cost** | | | | ¥ - | | | ¥ - | | | ¥ 67.36 | |  | | ¥ 124.13 | | | ¥ 184.52 | | | ¥ 246.60 |
| **75-84 years** | | | |  | | |  | | |  | |  | |  | | |  | | |  |
| **percent of all untreated HT** | | | | 26.4% | | | 9.1% | | | 29.0% | |  | | 13.2% | | | 12.2% | | | 10.1% |
| **percent of stage** | | | | 0.0% | | | 0.0% | | | 44.9% | |  | | 37.1% | | | 34.4% | | | 28.5% |
| **BP change after treatment** | | | | 0.0 | | | 0.0 | | | 8.4 | |  | | 18.2 | | | 27.6 | | | 36.6 |
| **RR CHD after treatment** | | | | 1.00 | | | 1.00 | | | 0.89 | |  | | 0.77 | | | 0.68 | | | 0.60 |
| **RR stroke after treatment** | | | | 1.00 | | | 1.00 | | | 0.87 | |  | | 0.74 | | | 0.63 | | | 0.55 |
| **Annual anti-HT medication cost** | | | | ¥ - | | | ¥ - | | | ¥ 67.36 | |  | | ¥ 124.13 | | | ¥ 184.52 | | | ¥ 246.60 |
| **Women** | |  | | | |  | | |  | |  | |  | | |  | | |  | |
|  | | Stage 1 (140-159 mm Hg) | | | | | | | | |  | | Stage 2 ( ≥ 160 mm Hg) | | | | | | | |
| **Systolic BP interval** | | 140-145 | | | | 146-148 | | | 149-159 | |  | | 160-169 | | | 170-179 | | | >=180 | |
|  | |  | | | |  | | |  | |  | |  | | |  | | |  | |
| **Number of standard dose drugs** | | 0.5 | | | | 1.0 | | | 2.0 | |  | | 3.0 | | | 3.5 | | | 4.0 | |
| **35-44 years** | |  | | | |  | | |  | |  | |  | | |  | | |  | |
| **percent of all untreated HT** | | 40.0% | | | | 12.8% | | | 24.5% | |  | | 14.4% | | | 3.6% | | | 4.7% | |
| **percent of stage** | | 51.8% | | | | 16.5% | | | 31.7% | |  | | 63.6% | | | 15.8% | | | 20.7% | |
| **BP change after treatment** | | 4.2 | | | | 8.4 | | | 18.2 | |  | | 27.6 | | | 36.6 | | | 42.0 | |
| **RR CHD after treatment** | | 0.89 | | | | 0.80 | | | 0.61 | |  | | 0.47 | | | 0.37 | | | 0.32 | |
| **RR stroke after treatment** | | 0.86 | | | | 0.74 | | | 0.52 | |  | | 0.37 | | | 0.27 | | | 0.22 | |
| **Annual anti-HT medication cost** | | ¥ 33.68 | | | | ¥ 67.36 | | | ¥ 124.13 | |  | | ¥ 184.52 | | | ¥ 246.60 | | | ¥ 269.43 | |
|  | |  | | | |  | | |  | |  | |  | | |  | | |  | |
| **45-54 years** | |  | | | |  | | |  | |  | |  | | |  | | |  | |
| **percent of all untreated HT** | | 25.1% | | | | 16.0% | | | 26.5% | |  | | 14.6% | | | 7.5% | | | 10.3% | |
| **percent of stage** | | 37.1% | | | | 23.7% | | | 39.2% | |  | | 45.1% | | | 23.0% | | | 31.9% | |
| **BP change after treatment** | | 4.2 | | | | 8.4 | | | 18.2 | |  | | 27.6 | | | 36.6 | | | 42.0 | |
| **RR CHD after treatment** | | 0.90 | | | | 0.82 | | | 0.65 | |  | | 0.52 | | | 0.42 | | | 0.37 | |
| **RR stroke after treatment** | | 0.88 | | | | 0.77 | | | 0.57 | |  | | 0.42 | | | 0.32 | | | 0.27 | |
| **Annual anti-HT medication cost** | | ¥ 33.68 | | | | ¥ 67.36 | | | ¥ 124.13 | |  | | ¥ 184.52 | | | ¥ 246.60 | | | ¥ 269.43 | |
|  | |  | | | |  | | |  | |  | |  | | |  | | |  | |
| **55-64 years** | |  | | | |  | | |  | |  | |  | | |  | | |  | |
| **percent of all untreated HT** | | 22.6% | | | | 14.9% | | | 21.6% | |  | | 19.3% | | | 8.8% | | | 12.7% | |
| **percent of stage** | | 38.2% | | | | 25.2% | | | 36.6% | |  | | 47.3% | | | 21.5% | | | 31.2% | |
| **BP change after treatment** | | 4.2 | | | | 8.4 | | | 18.2 | |  | | 27.6 | | | 36.6 | | | 42.0 | |
| **RR CHD after treatment** | | 0.91 | | | | 0.83 | | | 0.68 | |  | | 0.55 | | | 0.45 | | | 0.41 | |
| **RR stroke after treatment** | | 0.89 | | | | 0.79 | | | 0.60 | |  | | 0.46 | | | 0.36 | | | 0.31 | |
| **Annual anti-HT medication cost** | | ¥ 33.68 | | | | ¥ 67.36 | | | ¥ 124.13 | |  | | ¥ 184.52 | | | ¥ 246.60 | | | ¥ 269.43 | |
|  | |  | | | |  | | |  | |  | |  | | |  | | |  | |
| **65-74 years** | |  | | | |  | | |  | |  | |  | | |  | | |  | |
| **percent of all untreated HT** | | 18.5% | | | | 16.5% | | | 30.0% | |  | | 19.1% | | | 6.0% | | | 9.8% | |
| **percent of stage** | | 0.0% | | | | 0.0% | | | 46.2% | |  | | 54.6% | | | 17.3% | | | 28.1% | |
| **BP change after treatment** | | 0.0 | | | | 0.0 | | | 8.4 | |  | | 18.2 | | | 27.6 | | | 36.6 | |
| **RR CHD after treatment** | | 1.00 | | | | 1.00 | | | 0.86 | |  | | 0.72 | | | 0.61 | | | 0.52 | |
| **RR stroke after treatment** | | 1.00 | | | | 1.00 | | | 0.82 | |  | | 0.65 | | | 0.52 | | | 0.42 | |
| **Annual anti-HT medication cost** | | ¥ - | | | | ¥ - | | | ¥ 67.36 | |  | | ¥ 124.13 | | | ¥ 184.52 | | | ¥ 246.60 | |
|  | |  | | | |  | | |  | |  | |  | | |  | | |  | |
| **75-84 years** | |  | | | |  | | |  | |  | |  | | |  | | |  | |
| **percent of all untreated HT** | | 18.3% | | | | 12.6% | | | 26.6% | |  | | 16.5% | | | 12.9% | | | 13.1% | |
| **percent of stage** | | 0.0% | | | | 0.0% | | | 46.3% | |  | | 38.8% | | | 30.4% | | | 30.8% | |
| **BP change after treatment** | | 0.0 | | | | 0.0 | | | 8.4 | |  | | 18.2 | | | 27.6 | | | 36.6 | |
| **RR CHD after treatment** | | 1.00 | | | | 1.00 | | | 0.90 | |  | | 0.79 | | | 0.70 | | | 0.62 | |
| **RR stroke after treatment** | | 1.00 | | | | 1.00 | | | 0.89 | |  | | 0.77 | | | 0.67 | | | 0.59 | |
| **Annual anti-HT medication cost** | | ¥ - | | | | ¥ - | | | ¥ 67.36 | |  | | ¥ 124.13 | | | ¥ 184.52 | | | ¥ 246.60 | |
|  | |  | | | |  | | |  | |  | |  | | |  | | |  | |
