# Supplementary material for: The Cost-Effectiveness of Low-Cost Essential Antihypertensive Medicines for Hypertension Control in China: A Modelling Study
Source: PLoS Med. 2015 Aug 4;12(8):e1001860. doi: 10.1371/journal.pmed.1001860 (PMC4524696; doi:10.1371/journal.pmed.1001860)
Supplement: S6 Table — (DOCX) [file pmed.1001860.s008.docx]

**S1 Table 6. Distributions of main input parameters used in probabilistic sensitivity analyses**

| **Parameter** | **Distribution sampled** | **Main estimate** | **Measure of dispersion** | **Magnitude of dispersion** | **Sources for estimates** |
| --- | --- | --- | --- | --- | --- |
| **Systolic Hypertension** | Normal | Percent of population | Standard error (%) |  |  |
| **Stage 1 (140-159 mmHg)**  Men  35-44 years  45-54  55-64  65-74  75-84 |  | 7.1  13.8  21.7  25.6  25.6* |  | 0.6  1.0  1.4  2.0  2.0* | InterASIA Study[^20^](#_ENREF_20) |
| Women  35-44 years  45-54  55-64  65-74  75-84 |  | 4.8  13.3  19.4  28.5  28.5* |  | 0.5  1.0  1.3  2.1  2.1* |  |
| **Stage 2 (140-159 mmHg)**  Men  35-44 years  45-54  55-64  65-74  75-84 |  | 1.1  4.1  11.8  16.1  16.1* |  | 0.2  0.6  1.0  1.8  1.8* | InterASIA Study[^20^](#_ENREF_20) |
| Women  35-44 years  45-54  55-64  65-74  75-84 |  | 1.4  6.4  13.4  15.4  15.4* |  | 0.2  0.7  1.1  1.6  1.6* |  |
| **Stage 1 (140-159 mmHg)**  Men  35-44 years  45-54  55-64  65-74  75-84 | Normal | Mean (mmHg)  146.5  146.9  148.0  148.1  148.1* | Standard error (mmHg) | 0.5  0.5  0.4  0.5  0.5* | InterASIA Study[^20^](#_ENREF_20) |
| Women  35-44 years  45-54  55-64  65-74  75-84 |  | 146.1  148.1  147.4  149.4  149.4* |  | 0.6  1.5  1.1  1.3  1.3* |  |
| **Stage 2 (140-159 mmHg)**  Men  35-44 years  45-54  55-64  65-74  75-84 |  | 173.9  174.6  176.9  179.7  179.7* |  | 2.8  1.8  1.8  2.2  2.2* | InterASIA Study[^20^](#_ENREF_20) |
| Women  35-44 years  45-54  55-64  65-74  75-84 |  | 173.2  175.4  174.3  172.3  172.3* |  | 2.1  1.5  1.1  1.6  1.6* |  |
| **Hypertension treatment**  Men  35-44 years  45-54  55-64  65-74  75-84 | Normal | Percent of whole population  2.4  6.6  11.0  14.4  14.4 | Standard error (%) | 0.4  0.7  1.0  1.6  1.6 | InterASIA Study[^20^](#_ENREF_20) |
| Women  35-44 years  45-54  55-64  65-74  75-84 |  | 2.3  10.1  14.4  16.7  16.7* |  | 0.3  0.8  1.1  1.7  1.7* |  |
| **Case-fatality** | Normal | Percent of cases dying of any cause | 95% confidence interval |  |  |
| **Hospitalized acute myocardial Infarction**  Men  35-44 years  45-54  55-64  65-74  75-84 |  | 5.0  15.0  18.5  25.0  50.0 |  | 0.4  1.1  1.3  1.8  3.5 | Bridging the Gap in Acute Coronary Syndromes Study (Multi-provincial hospital based study)[^16^](#_ENREF_16)^,^ [^17^](#_ENREF_17) |
| Women  35-44 years  45-54  55-64  65-74  75-84 |  | 15.0  20.0  20.0  30.0  50.0 |  | 1.1  1.4  1.4  2.1  3.5 |  |
| **Acute stroke**  Men  35-44 years  45-54  55-64  65-74  75-84 | Normal | 25.0  18.0  12.0  20.0  45.0 |  | 0.4  0.4  0.4  0.4  0.4 | Sino-MONICA Beijing surveillance study[^18^](#_ENREF_18) |
| Women  35-44 years  45-54  55-64  65-74  75-84 |  | 18.0  14.0  15.0  20.0  45.0 |  | 0.6  0.6  0.6  0.6  0.6 |  |
| **Hospitalized acute CVD costs**  Hospitalized Stroke  Angina pectoris  Acute MI  Acute MI with percutaneous coronary intervention  Acute MI with coronary artery bypass graft surgery  Acute heart failure | Normal | 2010 Chinese RMB  8,015  7,887  15,774  39,434  80,671  8,885 | Standard deviation | 2,706  2,706  2,706  2,706  2,706  2,706 | National variation in hospital length of stay,  China PEACE Study[^19^](#_ENREF_19) |
| **Chronic CVD costs**  First year after MI  Subsequent years after MI  First year after stroke  Subsequent years after stroke | Normal | 2010 Chinese RMB  3,245  2,261  1,980  1,275 | 95% confidence interval | 67  47  41  26 | Variation in outpatient visit costs between rural and urban China, World Health Organization CHOICE regression analysis[^21^](#_ENREF_21) |
| **Blood pressure change** | Normal | See manuscript table 2 and Appendix table 10 | Standard deviation | 0.15 | Law and Wald trials meta-analysis (2003)[^22^](#_ENREF_22) |
| **Relative risk of CVD**  **Coronary heart disease**  Men  35-44 years  45-54  55-64  65-74  75-84 | Normal | Beta coefficients; Per 1 mmHg SBP or 0.5 mmHg DBP  0.0325  0.0310  0.0300  0.0265  0.0230 | 95% confidence interval | Per 1 mmHg SBP  or 0.5 mmHg DBP  0.00352  0.00163  0.00124  0.00174  0.00174 | Law, Morris, and Wald trials meta-analysis and Prospective Studies Collaboration  (2009)[^13^](#_ENREF_13)^,^ [^23^](#_ENREF_23) |
| Women  35-44 years  45-54  55-64  65-74  75-84 |  | 0.0320  0.0320  0.0295  0.0245  0.0228 |  | 0.00348  0.00144  0.00124  0.00165  0.00165 |  |
| **Stroke**  Men  35-44 years  45-54  55-64  65-74  75-84 |  | 0.0500  0.0460  0.0420  0.0370  0.0230 |  | 0.00472  0.00343  0.00214  0.00401  0.00218 |  |
| Women  35-44 years  45-54  55-64  65-74  75-84 |  | 0.0470  0.0450  0.0414  0.0345  0.0267 |  | 0.00476  0.00318  0.00220  0.00459  0.00208 |  |
| **Bundled treatment costs (medications, monitoring, and side effects)** | Normal | See manuscript table 2 | Coefficient of variation | 0.05 | Distribution of bundled costs when component costs were assembled (driven by medication costs; see Appendix C) |
| **Quality of life penalty** | Normal | See manuscript table 2 | Standard deviation | 0.258 | Side effect frequency in Law and Wald trials meta-analysis (2003)[^22^](#_ENREF_22) |
