# Supplementary material for: The Cost-Effectiveness of Low-Cost Essential Antihypertensive Medicines for Hypertension Control in China: A Modelling Study
Source: PLoS Med. 2015 Aug 4;12(8):e1001860. doi: 10.1371/journal.pmed.1001860 (PMC4524696; doi:10.1371/journal.pmed.1001860)
Supplement: S7 Table — (DOCX) [file pmed.1001860.s009.docx]

**S1Table 7. Cost effectiveness hypertension control in Chinese adults with and without adding separate components of program costs. All estimates are incremental cost-effectiveness ratios (ICERs), compared with the prior strategy. Results are in 2015 international dollars (2015 Chinese RMB). All results reported as cost-saving describe strategies projected to be less costly and more effective than the prior strategy.**

| Strategy | **Strategy 1:** Treat all stage two hypertension patients to goal of <140/90 mmHg if age 35-64 years, goal of 150/90 mmHg if age ≥65 in addition to CVD patients | **Strategy 2:** Treat stage two and stage one, goal <140/90 mmHg if age 35-64 years, goal of 150/90 mmHg if age ≥65, in addition to CVD patients |
| --- | --- | --- |
| Comparator for ICER | Treat only CVD patients (base case) | Strategy 1 |
| Hypertension treatment alone (costs of medications, monitoring, side effects only) | Cost-saving | $12,000 (¥ 42,200)§ |
| Add cost of systematic hypertension screening program in ages 35-84 years¶ | $8,200 (¥ 28,700)∆ | $12,000 (¥ 42,200)§ |
| Cost of implementing Essential Medicines use  Add 5% of drug expenditures | Cost-saving | $12,200 (¥ 43,000)§ |
| Add 15% of drug expenditures | $100 (¥ 200)∆ | $12,600 (¥ 44,500)§ |
| Cost of administering hypertension control program  Add 5% of total screening and treatment costs for program administration | $400 (¥ 1,500)∆ | $12,800 (¥ 45,000)§ |
| Include all added program costs (main analysis)  Add screening program, 15% of drug expenditures, and 5% program administration cost | $8,900 (¥ 31,300)∆ | $13,500 (¥ 47,400)§ |

¶Normotensive once every two years, prehypertensive once yearly

∆Less than 1 x China’s gross domestic product per capita

§Less than 2 x China’s gross domestic product per capita, but not less than 1 x GDP per capita
