# Supplementary material for: The Cost-Effectiveness of Low-Cost Essential Antihypertensive Medicines for Hypertension Control in China: A Modelling Study
Source: PLoS Med. 2015 Aug 4;12(8):e1001860. doi: 10.1371/journal.pmed.1001860 (PMC4524696; doi:10.1371/journal.pmed.1001860)

**S1 Text: Supplementary Material**

**Appendix A: Detailed description of the Cardiovascular Disease Policy Model-China**

**Overview**

The Cardiovascular Disease (CVD) Policy Model-China is a computer-simulation, state-transition (Markov cohort), mathematical model of coronary heart disease and stroke incidence, prevalence, mortality, and costs in the adult Chinese population (see **Figure, below**).[^1^](#_ENREF_1) The model start year is 2000 and the model cycle length is one year. Simulations are at the national population level. The standard model simulates a dynamic national population, adding waves of 35-year adults with each successive cycle. The model can be adapted to simulate a closed cohort (i.e., waves of younger adults are not added).

The CVD Policy Model consists of three submodels: the demographic-epidemiologic model, the bridge model and the disease history model. The Demographic-Epidemiologic Submodel predicts coronary heart disease and stroke incidence and non-CVD mortality among subjects without CVD, stratified by age, sex, and up to 8 additional categorized risk factors estimated in Chinese adults in ten-year age categories ages 35-84 years in the 2000-2002 International Collaborative Study of Cardiovascular Disease in Asia Study (InterASIA).[^2^](#_ENREF_2) Risk factors include: systolic blood pressure (<140, 140-159.9, ≥160 mmHg), isolated diastolic blood pressure (normal systolic blood pressure and diastolic blood pressure (90-99 or ≥100 mmHg), antihypertensive medication treatment status (self-reported), smoking status (active smoker, non-smoker with exposure to environmental tobacco smoke, non-smoker without environmental exposure), high density lipoprotein (HDL) cholesterol (<1.0, 1.0-1.5, ≥1.6 mmol/L; <40, 40-59.9, ≥60 mg/dL), low-density lipoprotein (LDL) cholesterol (<2.6, 2.6-3.3, ≥3.4 mmol/L; <100, 100-129.9, ≥130 mg/dL), body mass index (<25, 25-29.9, ≥30 kg/M^2^), diabetes mellitus (yes or no), and chronic kidney disease [estimated glomerular filtration rate of < 60 ml/min (using the Modification of Diet in Renal Disease formula)]. Mean blood pressure was the average of the second two of three seated measurements, measured after a five minute wait by a trained observer and using a standard and calibrated mercury sphygmomanometer and appropriate cuff size. Hypertension status and hypertension awareness were established based on self-reported physician diagnosis, report of taking anti-hypertensive drugs, and measured blood pressure. Age trends in risk factor levels were preserved over time.

After CVD develops, the Bridge Submodel characterizes the initial stroke or coronary heart disease event (cardiac arrest, myocardial infarction, or angina) and its sequelae for 30 days. Then, the Disease History Submodel predicts subsequent CVD events, coronary revascularization procedures, CVD mortality, and non-CVD mortality among patients with CVD, stratified by age, sex, and history of events. The general chronic CVD categories are coronary heart disease only, stroke only, and combined prior coronary heart disease and prior stroke. Each state and event has an annual cost and quality-of-life adjustment as well as an annual probability of a repeat event and/or transition to a different CVD state. There was an assumption that there is no remission to CVD-free state after incident CVD. All population distributions, risk factor levels, coefficients, event rates, case fatality rates, costs, and quality-of-life adjustments can be modified for forecasting simulations.

**Figure. CVD Policy Model-China structure.** State transitions are numbered in the diagram and transition probability functions are described mathematically in the next section.

Transition 1 = remain in CVD-free state. Transition 2 = incident CVD. Transition 3 = nonCVD death. Transitions 4 and 5 = survival or case-fatality. Transition 6 = survival with or without repeat CVD event in chronic CVD patients.


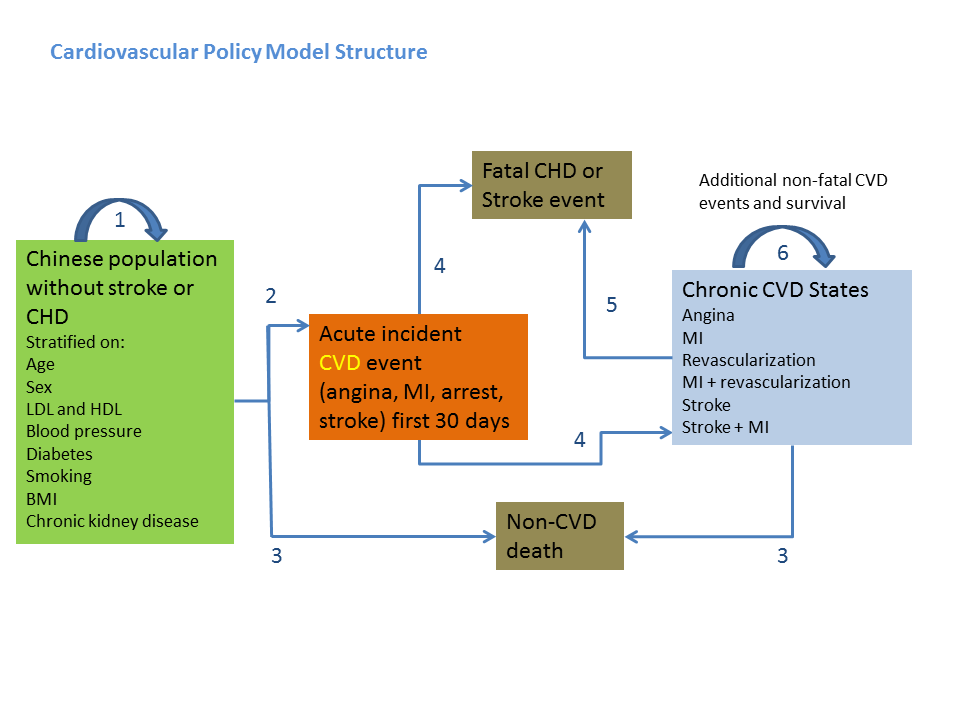


Stroke incidence,[^3^](#_ENREF_3)^,^ [^4^](#_ENREF_4) mortality,[^5^](#_ENREF_5) and case-fatality [^3^](#_ENREF_3) estimates were derived from other Chinese studies. The main outcomes predicted were CHD events (nonfatal and nonfatal first-ever and repeat episodes of stable and unstable angina, myocardial infarction, or cardiac arrest) and stroke events (nonfatal and fatal ischemic and hemorrhagic strokes). The CVD Policy Model-China defined coronary heart disease as myocardial infarction (ICD-9 410, 412 or ICD-10 I21, I22), angina and other coronary heart disease (ICD-9 411, 413 and 414, or ICD-10 I20, I23-I25), and a fixed proportion of “ill-defined” CVD coded events and deaths (ICD-9 codes 427.1, 427.4, 427.5, 428, 429.0, 429.1, 429.2, 429.9, 440.9 or ICD-10 I47.2, I49.0, I46, I50, I51.4, I51.5, I51.9, and I70.9).[^6^](#_ENREF_6) Stroke was defined by ICD-9 codes 430-438 (excluding transient ischemic attack) or ICD-10 I60-I69. In a calibration procedure, coronary heart disease and stroke incidence were adjusted to match with age- and sex-specific mortality targets within one percent. “Cardiovascular disease” was defined as combined CHD and total (ischemic + hemorrhagic) stroke.

**Mathematical Description of the CVD Policy Model**

P_k_ = the population in year k without CHD, distributed into 23,328 risk factor cells (23,328 x 1 vector)

D_k_ = the population in year k with CHD, distributed into 72 disease history cells (72 x 1)

P_0_ is determined from China Census and InterASIA survey data

D_0_ is determined (mostly) by CHEFS self-report data

The model is a discrete linear dynamical system described by the following equations:

P_k+1_ = Q(I-F1-F2)(I-F3) P_k_ + N35

B_k_=F1(I-F3) P_k_

C_k_=F2(I-F3) P_k_

D_k+1_ = S[(T-F4)D_k_ + UAB_k +_ VC_k_] +M35

where

I = nxn identity matrix, n = 23,328

F1 = nxn diagonal risk function matrix for incident coronary heart disease (China Multi-provincial cohort study; CMCS)

F2 = nxn diagonal risk function matrix for incident stroke (CMCS)

F3 = nxn diagonal risk function matrix for non-chd death (vital statistics less CMCS CVD deaths)

F4 = 72 x 72 diagonal non-CVD mortality rate for persons with prior CVD

B_k_ = n x 1 incident coronary heart disease population in year k (by DE risk cell):

C _k_ = n x 1incident stroke population in year k (by DE risk cell)

Annual probabilities of first CVD events or non-CVD deaths are determined by functions in the form of logistic regression equations:

$\frac{e^{\left( \alpha+\sum\beta_{RF}\times{MEAN}_{RF} \right)}}{1+e^{\left( \alpha+\sum\beta_{RF}\times{MEAN}_{RF} \right)}}$

where α = age-specific rate of disease in the overall population (intercept), β = age-specific risk coefficient, and RF = risk factor, and MEAN = risk factor mean exposure level.

D_k_ = chronic CVD survivor population (coronary heart disease, stroke, and coronary heart disease + stroke population)

T = a 72 x 72 block diagonal state transition matrix, with one 6x6 block for each of the 12 age-range/gender groups) for movement between the 6 disease history states, incorporating event rates and event survival rates.

A = the 48 x n allocation matrix, distributing incident coronary heart disease cases among the 4 incident CHD events (angina, AMI, AMI+revascularization, arrest) and incident total stroke events by age-range and gender, and including survival rates associated with each incident event. Survival rates are determined by event-, age-, and sex specific population-based one day and 30 day case-fatality (from the Sino-MONICA Beijing Study)

Case fatality = probability of death given an acute disease event = fatal cases/all cases

U = a 72 x 48 block diagonal state transition matrix for a second coronary heart disease or stroke event in the first year of incident CVD, including survival rates for each event

V = a 72 x n block diagonal transition matrix including incident stroke survival rates and subsequent rates of second CVD events and survival.

S and Q denote age shift operators that advance every person to the next age; in the non-CVD population, Q incorporates age-related transitions between risk factor levels

N35 = incoming 35 year olds without CVD, distributed into risk factor cells

M35 = incoming 35 year olds with CVD, distributed into 6 disease history states

Interventions:

- changes to risk factor means (e.g., lower blood pressure, as in this study) affect F1, F2 and F3
- changes to CVD event rates and case fatality rates affect T, A, U and V

**General CVD Risk Factor Effects**

Methods related to blood pressure-related inputs are reported below. For the standard China CVD Policy Model, annual probability of first CVD events and non-CVD deaths conditioned on demographic and risk factors were estimated by analyzing the China Multi-provincial Cohort Study (CMCS). The CMCS was a cohort study of 30,121 male and female participants aged 35-64 years and with no cardiovascular disease at baseline in 1992-1993 (more details about the CMCS provided in a manuscript by Liu et al).[^7^](#_ENREF_7) These participants were recruited from 16 centers in 11 Chinese provinces using a multistage sampling method. Twelve centers (80.3% of participants) were in urban areas and the remainder in rural areas. Overall baseline participation rate was 82%. Baseline measurement of risk factors using a standard protocol (WHO-MONICA protocol)[^8^](#_ENREF_8) and blood samples were processed at a central laboratory. Case-finding of new CHD and stroke events and non-cardiovascular deaths was first done by face-to-face interview. Events were ascertained by 1) detailed interview of participants or family members, 2) review of hospital records. These events were later adjudicated by investigators at the Beijing Institute for Heart, Lung, and Blood Vessel Diseases. After 1996, six centers ceased follow up because of completion of that national research project, but the remaining 10 centers (16 552 participants) were followed up through the end of 2002. Follow up rate was 86% for the centers followed all of 1992-2002, and 65% of the original 16 center cohort. Multivariate Cox proportional hazard ratios for systolic blood pressure (SBP), diabetes mellitus, total cholesterol, BMI, and active cigarette smoking were estimated from baseline measurements and ischemic and hemorrhagic events occurring over 159,400 person-years of observation in CMCS participants aged 35-74 years (appendix table 1).[^7^](#_ENREF_7) Significant (*P* < 0.05) age*risk factor risk coefficient interactions (higher risk at higher ages) were observed for smoking in CMCS multivariate CHD models, SBP, and smoking in total stroke models, and smoking and diabetes in non-cardiovascular mortality models, so these were incorporated in age-specific risk coefficients. Methods related to blood pressure beta coefficients are reported below.

**Epidemiologic input parameters**

Prior to calibration (see below), CHD incidence in men and women aged 35-84 years with no prior CHD diagnosis was based on 10-year incidence rates from the China Hypertension Epidemiology Follow Up Study (CHEFS)[^4^](#_ENREF_4) and calibrated to fit with CHD mortality and case-fatality assumptions. Incident stroke rates were also identified from the CHEFS.[^4^](#_ENREF_4) Main CHD Policy Model-China 28-day case-fatality assumptions were estimated from pooled Beijing Sino-MONICA Study data from 1993-2004 (personal communication, Dong Zhao, MD, PhD, 2006) and the main age-specific CHD case-fatality rate assumptions were estimated from the overall rates. Self-reported history of a physician-diagnosed myocardial infarction and/or stroke was based on data from CHEFS. In CHEFS, each self-reported case of prevalent CVD was ascertained by chart review by study staff. Final epidemiologic parameter estimates are show in **Appendix** **Tables A and B**.

**Appendix Table A. Coronary Heart Disease (CHD) Inputs used for the CVD Policy Model-China**

| **Sex/age** | **CHD incidence rate per 100,000** | **CHD 28 day case-fatality (proportion)** | **CHD Mortality**  **per 100,000** | **Prevalence of prior myocardial infarction (proportion)** |
| --- | --- | --- | --- | --- |
| Men  35-44 years  45-54  55-64  65-74  75-84 | 130  135  220  500  2,010 | 0.12  0.21  0.29  0.33  0.48* | 10  36  97  243  1,104 | 0.006  0.012  0.034  0.047  0.060* |
| Women  35-44 years  45-54  55-64  65-74  75-84 | 19  49  141  310  1,900 | 0.18  0.23  0.27  0.43  0.51* | 1  20  43  160  1,028 | 0.004  0.013  0.031  0.040  0.060* |

*Estimate not available from original source data and imputed using linear interpolation

**Appendix Table B. Stroke Inputs used for the CVD Policy Model-China**

| **Sex/age** | **Total stroke Incidence rate/100,000** | **Total stroke 28 day case-fatality (proportion)** | **Total stroke Mortality/100,000** | **Prevalence of prior stroke**  **(proportion)** |
| --- | --- | --- | --- | --- |
| Men  35-44 years  45-54  55-64  65-74  75-84 | 24  145  670  1,250  2,510 | 0.25  0.18  0.12  0.20  0.45* | 20  62  151  502  1,708 | 0.013  0.032  0.088  0.142  0.150* |
| Women  35-44 years  45-54  55-64  65-74  75-84 | 23  180  800  1,500  2,500 | 0.18  0.14  0.15  0.20  0.45* | 10  30  131  375  1,359 | 0.009  0.024  0.060  0.100  0.120* |

*Estimate not available from original source data and imputed using linear interpolation

**Model calibration: comparison with mortality rate targets for China**

In order to evaluate the accuracy of CVD Policy Model predictions over time, China stroke and CHD mortality estimates for ages 35-84 years were obtained from the China Ministry of Health (MOH) and the World Health Organization (WHO). In the calibration procedure, CHD and stroke parameters were calibrated separately. Starting with default incidence, case-fatality, and prevalence assumptions, the simulation model was run forward from year 2000 to 2010, and incidence inputs were iteratively calibrated primarily to match with age-specific WHO mortality numbers in 2010 overall and within ten-year age groups. A secondary aim was to remain consistent with WHO and China MOH time trends over the period 2000-2010.

**Appendix Table C. Precalibration and postcalibration CVD incidence inputs**

| **Sex/age** | **Total stroke Incidence rate/100,000** | | **CHD incidence rate per 100,000** | |
| --- | --- | --- | --- | --- |
|  | Pre-calibration (based on CHEFS, 1991-2000, ICD9 430-438) | Post-calibration (identical CVDPM definition) | Pre-calibration (based on CHEFS, 1991-2000, limited to ICD9 410-414) | Post-calibration (broader CVD Policy Model definition) |
| Men  35-44 years  45-54  55-64  65-74  75-84 | 91  240  711  1,292  1,904 | 24  145  670  1,250  2,510 | 54  112  342  540  889 | 130  135  220  500  2,010 |
| Women  35-44 years  45-54  55-64  65-74  75-84 | 59  176  424  848  1,500 | 23  180  800  1,500  2,500 | 23  96  188  368  752 | 19  49  141  310  1,900 |

Post-calibration CVD mortality predictions were reasonably close to WHO estimates for 2010: CHD mortality was about 5% lower than the WHO estimate, and stroke mortality was about 1% higher. In terms of trend, CHD mortality followed both WHO and China MOH reasonably well (**Figures below**). Stroke mortality numbers did not follow the diminishing trend over 2000-2010 suggested by the WHO data (**Figures below**). This is because the CVD Policy Model simulation did not assume any changes in case-fatality, disease incidence or risk factor exposure levels over the interval—therefore, the only driver of mortality numbers was population aging.

**Figures. Projected CVD deaths, China, compared with observed deaths, 1985-2010**

The CVD Policy Model can simulate trends in any of case-fatality, risk factors, or incidence, but because this analysis focused on hypertension treatment over a short time horizon (ten-years), we took a conservative approach and assumed no change in these factors over the simulation period.

After CVD incidence and mortality were satisfactorily calibrated, age and sex specific non-CVD death rates were calibrated so that the totals of CVD and non-CVD deaths fit within the envelope of all-cause or total mortality estimated for China by the WHO for 2008 (highest priority for calibration) and 2004 (secondary priority for calibration). The result was that 2008 total mortality for males and females ages 35-84 years was within 1% of the WHO 2008 estimate and within 3% for males and 6% for females compared with the 2004 WHO estimate (**Appendix Table D** below).

**Appendix Table D. Total deaths projected by the CVD Policy Model and observed by the WHO (simulated data are rounded to the 1,000s digit).**

| **China CVDPM total deaths, projected** | | |  |  | **WHO target, total deaths, observed** | | |
| --- | --- | --- | --- | --- | --- | --- | --- |
| year | both (35-84) | males (35-84) | females (35-84) |  | both (35-84) | males (35-84) | females (35-84) |
| **2004** | 6,720,000 | 3,850,000 | 2,869,000 |  | 7,039,014 | 3,999,893 | 3,039,121 |
| **2008** | 7,472,000 | 4,293,000 | 3,178,000 |  | 7,417,749 | 4,253,932 | 3,163,818 |

For an exploratory analysis, we projected the main hypertension control program simulation after calibrating the model (increasing incidence and prevalence) to reproduce China MOH deaths for 2000-2007. Below in Appendix Table 4 are the stroke and CHD incidence rates used for the China MOH and main (WHO) incidence assumptions.

**Appendix Table E. Incidence rates assumed for the main simulations (WHO mortality target; see Appendix Table C above) and the higher incidence based on higher China Ministry of Health mortality estimate targets.**

| **Sex/age** | **Total stroke Incidence rate/100,000** | | **CHD incidence rate per 100,000** | |
| --- | --- | --- | --- | --- |
|  | Incidence calibrated to match China Ministry of Health target | Main CVD Policy Model-China input (see Appendix Table 2) | Incidence calibrated to match China Ministry of Health target | Main CVD Policy Model-China input (see Appendix Table 2) |
| Men  35-44 years  45-54  55-64  65-74  75-84 | 31  210  1,000  1,950  5,500 | 24  145  670  1,250  2,510 | 137  148  225  670  2,700 | 130  135  220  500  2,010 |
| Women  35-44 years  45-54  55-64  65-74  75-84 | 31  163  453  1,231  5,300 | 23  105  355  830  2,500 | 28  60  141  340  2,550 | 19  49  141  310  1,900 |

**Quality-adjusted life year (QALY) adjustments for CVD states**

Life-years were tabulated for the population alive in each model cycle. Quality-adjusted life-years accounted for symptoms and disability that lead to less-than-perfect health in CVD patients. QALY weights were calculated as 1−disability-adjusted life year weights which were estimated in the Global Burden of Disease 2010 Study (**Appendix table F**).[^9^](#_ENREF_9) The annual number of QALYs gained due to salt restriction was calculated by subtracting the total QALYs after intervention from the total before the intervention.

**Appendix table F. QALYs weights calculated** **from Global Burden of Disease 2010 Study estimates**[**^10^**](#_ENREF_10)

|  | **The first 30 days after onset** | **Chronic State (after day 30)** |
| --- | --- | --- |
| **Nonfatal AMI** | 0·9520 | 0·9648 |
| **Nonfatal angina** | 0·9520 | 0·9604 |
| **Nonfatal stroke** | 0·8644 | 0·8835 |
| **Death** | 0·0000 | 0·0000 |

QALYs = quality-adjusted life years, calculated as (1-disability-adjusted life year weight)

AMI = acute myocardial infarction.

**Cardiovascular disease costs in China**

The cost included background health care costs, acute treatment costs, and chronic state costs of CVD which were estimated from a care system payer’s perspective in this study (**Appendix table G**). The annual cost for the chronic CVD states (stroke and coronary heart disease) were collected in 2008 and inflated to 2010 using the average rate of inflation in China from 2009 to 2010 published by Trading Economics. All CVD Policy Model-China costs data are entered into the model and calculated in 2010 Chinese RMB. For the manuscript, costs were converted into US dollars ($) using the purchasing power parity (PPP) conversion rate published by the World Bank. To convert cost inputs or results back to Chinese currency, multiply by purchasing power parity (PPP) rate (in this case, 3.52). To convert costs reported in the manuscript to $US using the current official exchange rate, multiply by (PPP/exchange rate), for example 3.52/6.20, or by 5.68.

**Appendix table G. Health care costs in China (2010 costs)**

| **Category** | **Costs($)** | **Year of Data Collection** | **Source and Publication Year** |
| --- | --- | --- | --- |
| **Hospital charges** |  |  |  |
| AMI | 3 943 | 2010 | China Public Health Statistical Yearbook (2011) [^11^](#_ENREF_11) |
| Stroke | 2 004 | 2010 |  |
| Hemorrhage stroke | 2 755 | 2010 |  |
| Ischemic stroke | 1 786 | 2010 |  |
| **Annual outpatient coronary heart disease** |  |  |  |
| The first year | 811 | 2008 | Initiative for Cardiovascular Health (IC-Health) Research and inflated to 2010 [^12^](#_ENREF_12) |
| After the first year | 565 | 2008 |  |
| **Annual outpatient stroke** |  |  |  |
| The first year | 495 | 2008 | Initiative for Cardiovascular Health (IC-Health) Research and inflated to 2010 |
| After the first year | 319 | 2008 |  |
| **Per capita total expenditure on health** | 219 | 2010 | World Health Statistics 2013 |

AMI, acute myocardial infarction; CHD, coronary heart disease.

**Appendix B: Additional material on methods for the analysis of hypertension treatment**

Starting with CVD Policy Model default blood pressure beta coefficients estimated from CMCS data, we calibrated blood pressure coefficients in order to 1) reproduce the association of change in systolic BP estimated from both the Prospective Cohort Studies Collaborative, and 2) a large pooled analysis of BP treatment trials by Law, Morris, and Wald.[^13^](#_ENREF_13) A cohort representing the age and sex structure and mean systolic BP of the stage one and stage two hypertensive population was prepared for calibration simulations. In order to simulate the average trial pooled in the Law, Morris and Wald study, five year treatment duration was simulated. First, a base case was simulated or 2010-2014 with no change in 2010 blood pressure levels. A 2010-2014 intervention simulation followed in which systolic BP was lowered by approximately 10 mm Hg or diastolic blood pressure was lowered 5 mm Hg in each age and sex category. Beta coefficients were calibrated until the Prospective Cohort Studies Collaboration age- and sex- specific relative risks were matched within 0.02 or less (**Appendix Table 1**). Summary (age and sex weighted) relative risk with a 10 mm Hg systolic or 5 mm Hg diastolic blood pressure change for ages 35-74 years of 0.75 was within the 95% confidence limits of the treatment trials relative risks estimated in the Law, Morris, and Wald meta-analysis for coronary heart disease (target interval 0.73-0.83), as was the estimate for stroke (estimate 0.64; target interval 0.52-0.67; **Appendix Table 2**).

In order to ensure that the systolic BP relative risk inputs were not over-fitted to the calibration targets, and not representative of the results of a real clinical trial, we set out to use the model to simulate the landmark Systolic Hypertension in the Elderly Program (SHEP), a double-blind placebo-controlled trial of antihypertensive adults aged 60 and older.   By analyzing individual participant-level data, we were able to enter the characteristics of the SHEP intervention arm and control arms (age, baseline systolic BP, mean HDL and LDL cholesterol, and smoking status of participants).  We then simulated the SHEP trial over a five-year period (mean follow up in SHEP lasted 4.5 years).  Cox proportional hazards analysis of SHEP was performed in order to ensure that we could reproduce the estimates reported in the 1999 SHEP trial report, and we performed original analyses of SHEP data in order estimate the total stroke (fatal and nonfatal), and total and fatal coronary heart disease relative risks associated with BP treatment consistent with CVD Policy Model definitions.

For each of stroke and CHD, we simulated RRs of stroke and CHD, assuming baseline characteristics of the SHEP cohort and systolic blood pressure beta coefficients used in our main analysis (these based on the Prospective Cohort Studies Collaboration and Law, Morris, Wald meta-analysis of trials as described above). Stroke and CHD RRs resulting from the simulated trial were then compared with the main estimates and 95% confidence intervals of the observed effects reported by the SHEP trial (**Appendix Table 8**).

Compared with the results observed in the actual trial, our five-year simulation of the SHEP trial resulted in nearly perfectly matched results for reductions in the rate of coronary heart disease events with treatment. For stroke, our simulations yielded a 30% reduction in strokes (relative risk 0.70) compared with the 36% reduction observed in the trial (relative risk 0.64).  SHEP excluded potential participants if they had a history of atrial fibrillation, but our Model  cannot selectively do so.  Since five-year effects of antihypertensive treatment are unlikely to lower risk for stroke caused by atrial fibrillation, the inclusion of people with atrial fibrillation likely explains why our simulated reduction of nonfatal stroke was less than that observed in SHEP. If we adjust our total stroke relative risk for an assumed 15% of all strokes (fatal and nonfatal) due to atrial fibrillation,[^14^](#_ENREF_14) and unaffected by the BP lowering intervention, our simulated trial would yield a relative risk of total stroke of 0.66 (calculated as:  exp[1.15*ln(0.70)]), very close to the SHEP total stroke relative risk of 0.64.

We did not choose to simulate the effects of any particular medication; instead we simulate “standard dose” effects and assumed average drug prices across classes. Examples of standard doses for different antihypertensive medications can be found at <http://www.wolfson.qmul.ac.uk/bpchol/a3.pdf>, and include nifedipine 30 mg daily, amlodipine 5 mg daily, lisinopril 10 mg daily, hydrochlorothiazide 25 mg daily, and losartan 50 mg daily, Especially in patients with higher BPs (e.g. stage two hypertension), medications are added sequentially and BP lowered gradually over time. The amount of blood pressure change was assumed to be a function of the pre-treatment or baseline BP and the effect of a standard-dose antihypertensive agent at that pre-treatment level (**Appendix Tables 9 and 10**). It is important to note that for patients with very high BPs (mean systolic BP of 185 mm Hg or more) it was assumed that even with taking four standard dose medications, these patients would on average achieve a BP of about 143 mm Hg, near, but not at, the target of 140 mmHg. Thus, some patients were assumed to be “truly resistant” hypertensives.

**Probabilistic Sensitivity analysis methods**

The CVD Policy Model has a multi-input probabilistic sampling function that allows generation of probabilistic uncertainty output. The probabilistic (Monte Carlo) simulation sampled across uncertainty distributions of both general epidemiological and cost inputs and hypertension treatment program-specific inputs. **Appendix Table 12** shows the distribution types chosen for each parameter sampled. Magnitudes of dispersion about the main input were based on the source data as referenced in the table. In terms of general epidemiologic inputs, probabilistic analyses sampled InterASIA national survey distributions (standard errors) of mean systolic blood pressure, stage one and stage two hypertension prevalence, and use of antihypertensive drugs in the Chinese adult population after accounting for the multi-stage sampling strategy of the survey.[^2^](#_ENREF_2)^,^ [^15^](#_ENREF_15) For case-fatality, 95% confidence intervals for 28-day case fatality were obtained from the China BRIG-ACS Study for acute hospitalized acute myocardial infarction,[^16^](#_ENREF_16)^,^ [^17^](#_ENREF_17) and from the Beijing Sino-MONICA Study for acute stroke.[^18^](#_ENREF_18) The hospital-level standard deviation in CVD hospitalization costs was estimated based on the interquartile range of hospital days for acute coronary syndrome patients in secondary and tertiary level Chinese hospitals nationally in 2011 (six hospital days),[^19^](#_ENREF_19) assuming a normal distribution in hospital costs, and the formula [interquartile range/1.349]. This resulted in a standard deviation of 4.4 hospital days that was multiplied by the WHO CHOICE secondary and tertiary hospital day costs; a standard deviation of 2,706 Chinese RMB. 95% confidence intervals around chronic CVD costs were based on the difference between urban and rural hospital clinics in the WHO CHOICE unit costs regression analysis for China. In terms of the hypertension control program, the probabilistic analysis sampled distributions of antihypertensive drug BP-lowering effectiveness, CVD relative risk reduction with treatment, quality of life penalties and costs related to side effects, and drug and monitoring costs. Uncertainty distributions were randomly sampled 1,000 times with replacement, and 95% uncertainty intervals were calculated.

**S1 Table 1. Results of the systolic blood pressure calibration exercise. CMCS = China Multi-provincial Cohort Study. PSC = Prospective Studies Collaboration. CVDPM = CVD Policy Model inputs and simulation outputs. NA = not available from data source. Due to limited data availability for ages 85-94 years, the age 75-84 estimate was used for that group.**

|  | | Beta coefficients (per 1.0 mm Hg systolic blood pressure or 0.5 mm Hg diastolic blood pressure) | | | | | | Relative risks (after systolic blood pressure change of 10 mm Hg or diastolic blood pressure change of 5 mm Hg) | | | | | |
| --- | --- | --- | --- | --- | --- | --- | --- | --- | --- | --- | --- | --- | --- |
|  | | CHD | | | Stroke | | | CHD | | | Stroke | | |
| Age/sex category | Mean change in SBP (mm Hg) | CMCS  default | PCS target | CVDPM  (input) | CMCS  default | PCS target | CVDPM  (input) | CMCS  default | PCS target | CVDPM (output) | CMCS  default | PCS target | CVDPM (output) |
| Males |  |  |  |  |  |  |  |  |  |  |  |  |  |
| 35-44 | 9.57 | .0150 | .0361 | .0325 | .0310 | .0513 | .0500 | .86 | .71 | .73 | .73 | .61 | .60 |
| 45-54 | 10.38 | .0150 | .0353 | .0310 | .0310 | .0496 | .0460 | .86 | .70` | .72 | .73 | .61 | .59 |
| 55-64 | 10.38 | .0150 | .0330 | .0300 | .0310 | .0453 | .0420 | .86 | .71 | .73 | .73 | .63 | .62 |
| 65-74 | 10.88 | .0150 | .0285 | .0265 | .0310 | .0385 | .0370 | .86 | .74 | .76 | .73 | .66 | .66 |
| 75-84 | 11.58 | NA | .0228 | .0230 | NA | .0273 | .0267 | NA | .77 | .77 | NA | .74 | .73 |
| Females |  |  |  |  |  |  |  |  |  |  |  |  |  |
| 35-44 | 9.87 | 0.110 | .0360 | .0320 | .0260 | .0513 | .0470 | .90 | .70 | .72 | .77 | .60 | .60 |
| 45-54 | 10.35 | 0.110 | .0353 | .0320 | .0260 | .0496 | .0450 | .90 | .70 | .72 | .77 | .61 | .60 |
| 55-64 | 10.94 | 0.110 | .0330 | .0295 | .0260 | .0452 | .0414 | .90 | .70 | .72 | .77 | .62 | .61 |
| 65-74 | 11.68 | 0.110 | .0285 | .0245 | .0260 | .0385 | .0345 | .90 | .73 | .75 | .77 | .65 | .65 |
| 75-84 | 12.67 | NA | .0228 | .0228 | NA | .0269 | .0267 | NA | .76 | .75 | NA | .72 | .71 |

**S1Table 2. Comparison of CVD Policy Model trial simulation to Law Morris Wald meta-analysis of anti-hypertensive medication treatment trials, both assuming a systolic blood pressure change of 10 mm Hg or a diastolic blood pressure change of 5 mm Hg.**

| Outcome | Law, Morris, and Wald meta-analysis estimate | CVD Policy Model main estimate  Males | CVD Policy Model main estimate  Females |
| --- | --- | --- | --- |
| Coronary heart disease | 0.78 (0.73—0.83) | Ages 35-74 years: 0.75  Ages 35-64 years: 0.74 | Ages 35-74 years: 0.74  Ages 35-64 years: 0.73 |
| Stroke | 0.59 (0.52—0.67) | Ages 35-74 years: 0.64  Ages 35-64 years: 0.62 | Ages 35-74 years: 0.63  Ages 35-64 years: 0.62 |

**S1Table 3. Results of a simulated trial of blood pressure treatment in participants in the Systolic Hypertension in the Elderly Program (SHEP) trial, using relative risk inputs and transition probabilities in the CVD Policy Model version used for the analysis.**

| **SHEP Population RR (SBP lowering of 12 mmHg for 5 years)** | | |
| --- | --- | --- |
| Outcome | Actual SHEP paper RR and 95% CI | CVD Policy Model five year simulation results with PSC calibrated SBP betas [overall rate ratio (95% uncertainty interval)*] |
| Incident stroke | 0.64 (0.50-0.82) | 0.70 (0.66-0.75) |
| Incident coronary heart disease† | 0.75 (0.60-0.94) | 0.76 (0.73-0.79) |

*95% uncertainty intervals derived from 1,000 probabilistic simulations that sampled from within the 95% confidence intervals of age- and sex-specific Prospective Cohorts Studies Collaboration beta coefficients.

†SHEP incident coronary heart disease defined as first-in-trial nonfatal acute myocardial infarction, coronary revascularization, or coronary heart disease death. CVD Policy Model incident coronary heart disease defined as first-ever nonfatal acute myocardial infarction, angina pectoris with or without coronary revascularization, or coronary heart disease death.

**S1 Table 4. Sequential changes in blood pressure with successive standard dose medications, based on the trials-based blood pressure change prediction formulas of Law, Morris, and Wald.** For one standard-dose medication, the formula for calculating the change in systolic blood pressure was [9.1+0.10×(BS-154)], and the formula for calculating the change in diastolic blood pressure was [5.1+0.11×(BD-97)], in which BS denotes the baseline systolic blood pressure and BD the baseline diastolic blood pressure. The formula for one half-standard dose was [6.7+0.078 x (BS-150)] for systolic and [3.7+0.088 x (BD-90)] for diastolic.

|  | Mean start BP | BP change 1 drug | new BP | BP change 2nd drug | new BP | BP change 3rd drug | new BP | BP change 4th drug | new BP | Final BP change | |
| --- | --- | --- | --- | --- | --- | --- | --- | --- | --- | --- | --- |
| **Systolic blood pressure (mm Hg changes)** |  |  |  |  |  |  |  |  |  | **< 60 years old** | **≥ 60 years old** |
| effect of 4 std dose | 185 | 12.2 | 172.8 | 11.0 | 161.8 | 9.9 | 151.9 | 8.9 | 143.0 | 42.0 | 33.1 |
| effect of 3.5 std doses | 175 | 11.2 | 163.8 | 10.1 | 153.7 | 9.1 | 144.6 | 6.3 | 138.4 | 36.6 | 21.3 |
| effect of 3 std doses | 165 | 10.2 | 154.8 | 9.2 | 145.6 | 8.3 | 137.4 |  |  | 27.6 | 19.4 |
| effect of 2 std doses | 155 | 9.2 | 145.8 | 8.3 | 137.5 |  |  |  |  | 17.5 |  |
| effect of 1 std dose | 155 | 9.2 | 145.8 |  |  |  |  |  |  | 9.2 |  |
| effect of 1 std dose | 147 | 8.4 | 138.6 |  |  |  |  |  |  | 8.4 |  |
| effect of 0.5 std dose | 142 | 6.1 | 135.9 |  |  |  |  |  |  | 6.1 |  |
| effect of 0.5 std dose | 155 | 7.1 | 147.9 |  |  |  |  |  |  |  | 7.1 |
| **Diastolic blood pressure (mm Hg changes)** |  |  |  |  |  |  |  |  |  | **All ages** | |
| effect of 3 std doses | 107 | 6.6 | 100.5 | 5.9 | 94.6 | 5.2 | 89.4 |  |  | 17.7 |  |
| effect of 3 std doses | 104 | 6.3 | 97.7 | 5.6 | 92.1 | 5.0 | 87.2 |  |  | 16.8 |  |
| effect of 3 std doses | 105 | 6.3 | 98.2 | 5.6 | 92.5 | 5.0 | 87.5 |  |  | 17.0 |  |
| effect of 3 std doses | 105 | 6.4 | 98.6 | 5.7 | 92.9 | 5.1 | 87.9 |  |  | 17.1 |  |
| effect of 3 std doses | 103 | 6.2 | 97.2 | 5.5 | 91.7 | 4.9 | 86.8 |  |  | 16.6 |  |
| effect of 3 std doses | 107 | 6.6 | 100.5 | 5.9 | 94.6 | 5.2 | 89.4 |  |  | 17.7 |  |
| effect one std dose | 94 | 5.2 | 89.0 |  |  |  |  |  |  | 5.2 |  |
| effect one std dose | 94 | 5.2 | 88.8 |  |  |  |  |  |  | 5.2 |  |
| effect one std dose | 93 | 5.1 | 88.0 |  |  |  |  |  |  | 5.1 |  |
| effect of 0.5 std dose | 94 | 4.1 | 90.1 |  |  |  |  |  |  | 4.1 |  |
| effect of 0.5 std dose | 94 | 4.1 | 89.9 |  |  |  |  |  |  | 4.1 |  |

**S1 Table 5. Detailed effectiveness and drug cost assumptions for systolic BP (age, sex, BP interval). Effects reported in this table reflect 75% adherence to medications.**

| **Men** |  | | | |  | | |  | | | |  | |  |  | | |  | | |
| --- | --- | --- | --- | --- | --- | --- | --- | --- | --- | --- | --- | --- | --- | --- | --- | --- | --- | --- | --- | --- |
|  | Stage 1 (140-159 mm Hg) | | | | | | | | | | |  | | Stage 2 ( ≥ 160 mm Hg) | | | | | | |
|  |  | | | | | |  | | |  | |  | |  | | |  | |  | |
|  |  | | | | | |  | | |  | |  | |  | | |  | |  | |
| **Systolic BP interval** | 140-145 | | | | | | 146-148 | | | 149-159 | |  | | 160-169 | | | 170-179 | | >=180 | |
|  |  | | | | | |  | | |  | |  | |  | | |  | |  | |
| **Number of standard dose drugs** | 0.5 | | | | | | 1.0 | | | 2.0 | |  | | 3.0 | | | 3.5 | | 4.0 | |
| **35-44 years** |  | | | | | |  | | |  | |  | |  | | |  | |  | |
| **percent of all untreated HT** | | | 41.1% | | | | 20.0% | | | 25.4% | |  | | 7.6% | | | 1.5% | | 4.4% | |
| **percent of stage** | | | 47.5% | | | | 23.1% | | | 29.4% | |  | | 56.2% | | | 11.0% | | 32.8% | |
| **BP change after treatment** | | | 4.2 | | | | 8.4 | | | 18.2 | |  | | 27.6 | | | 36.6 | | 42.0 | |
| **RR CHD after treatment** | | | 0.89 | | | | 0.79 | | | 0.61 | |  | | 0.47 | | | 0.37 | | 0.32 | |
| **RR stroke after treatment** | | | 0.86 | | | | 0.75 | | | 0.53 | |  | | 0.38 | | | 0.28 | | 0.23 | |
| **Annual anti-HT medication cost** | | | ¥ 33.68 | | | | ¥ 67.36 | | | ¥ 124.13 | |  | | ¥ 184.52 | | | ¥ 246.60 | | ¥ 269.43 | |
|  | | |  | | | |  | | |  | |  | |  | | |  | |  | |
| **45-54 years** |  | | | | | |  | | |  | |  | |  | | |  | |  | |
| **percent of all untreated HT** | | | 35.3% | | | | 16.3% | | | 25.4% | |  | | 10.1% | | | 4.3% | | 8.6% | |
| **percent of stage** | | | 45.8% | | | | 21.2% | | | 33.0% | |  | | 44.0% | | | 18.8% | | 37.2% | |
| **BP change after treatment** | | | 4.2 | | | | 8.4 | | | 18.2 | |  | | 27.6 | | | 36.6 | | 42.0 | |
| **RR CHD after treatment** | | | 0.89 | | | | 0.80 | | | 0.61 | |  | | 0.47 | | | 0.37 | | 0.32 | |
| **RR stroke after treatment** | | | 0.86 | | | | 0.75 | | | 0.53 | |  | | 0.38 | | | 0.28 | | 0.23 | |
| **Annual anti-HT medication cost** | | | ¥ 33.68 | | | | ¥ 67.36 | | | ¥ 124.13 | |  | | ¥ 184.52 | | | ¥246.60 | | ¥ 269.43 | |
|  | | |  | | | |  | | |  | |  | |  | | |  | |  | |
| **55-64 years** |  | | | | | |  | | |  | |  | |  | | |  | |  | |
| **percent of all untreated HT** | | | 20.6% | | | | 18.2% | | | 25.9% | |  | | 16.2% | | | 9.1% | | 9.9% | |
| **percent of stage** | | | 31.8% | | | | 28.2% | | | 40.0% | |  | | 45.9% | | | 25.9% | | 28.2% | |
| **BP change after treatment** | | | 4.2 | | | | 8.4 | | | 18.2 | |  | | 27.6 | | | 36.6 | | 42.0 | |
| **RR CHD after treatment** | | | 0.90 | | | | 0.81 | | | 0.63 | |  | | 0.49 | | | 0.39 | | 0.34 | |
| **RR stroke after treatment** | | | 0.88 | | | | 0.78 | | | 0.58 | |  | | 0.44 | | | 0.34 | | 0.29 | |
| **Annual anti-HT medication cost** | | | ¥ 33.68 | | | | ¥ 67.36 | | | ¥ 124.13 | |  | | ¥ 184.52 | | | ¥246.60 | | ¥ 269.43 | |
|  | | |  | | | |  | | |  | |  | |  | | |  | |  | |
| **65-74 years** |  | | | | | |  | | |  | |  | |  | | |  | |  | |
| **percent of all untreated HT** | | 23.0% | | | | | 9.4% | | | 29.0% | |  | | 14.6% | | | 7.7% | | 16.3% | |
| **percent of stage** | | 0.0% | | | | | 0.0% | | | 47.2% | |  | | 37.9% | | | 19.9% | | 42.1% | |
| **BP change after treatment** | | 0.0 | | | | | 0.0 | | | 8.4 | |  | | 18.2 | | | 27.6 | | 36.6 | |
| **RR CHD after treatment** | | 1.00 | | | | | 1.00 | | | 0.85 | |  | | 0.71 | | | 0.59 | | 0.50 | |
| **RR stroke after treatment** | | | | 1.00 | | | 1.00 | | | 0.81 | |  | | 0.63 | | | 0.50 | | | 0.40 |
| **Annual anti-HT medication cost** | | | | ¥ - | | | ¥ - | | | ¥ 67.36 | |  | | ¥ 124.13 | | | ¥ 184.52 | | | ¥ 246.60 |
|  | | | |  | | |  | | |  | |  | |  | | |  | | |  |
| **75-84 years** | | | |  | | |  | | |  | |  | |  | | |  | | |  |
| **percent of all untreated HT** | | | | 26.4% | | | 9.1% | | | 29.0% | |  | | 13.2% | | | 12.2% | | | 10.1% |
| **percent of stage** | | | | 0.0% | | | 0.0% | | | 44.9% | |  | | 37.1% | | | 34.4% | | | 28.5% |
| **BP change after treatment** | | | | 0.0 | | | 0.0 | | | 8.4 | |  | | 18.2 | | | 27.6 | | | 36.6 |
| **RR CHD after treatment** | | | | 1.00 | | | 1.00 | | | 0.89 | |  | | 0.77 | | | 0.68 | | | 0.60 |
| **RR stroke after treatment** | | | | 1.00 | | | 1.00 | | | 0.87 | |  | | 0.74 | | | 0.63 | | | 0.55 |
| **Annual anti-HT medication cost** | | | | ¥ - | | | ¥ - | | | ¥ 67.36 | |  | | ¥ 124.13 | | | ¥ 184.52 | | | ¥ 246.60 |
|  | | | |  | | |  | | |  | |  | |  | | |  | | |  |
| **Women** | |  | | | |  | | |  | |  | |  | | |  | | |  | |
|  | | Stage 1 (140-159 mm Hg) | | | | | | | | |  | | Stage 2 ( ≥ 160 mm Hg) | | | | | | | |
|  | |  | | | |  | | |  | |  | |  | | |  | | |  | |
|  | |  | | | |  | | |  | |  | |  | | |  | | |  | |
| **Systolic BP interval** | | 140-145 | | | | 146-148 | | | 149-159 | |  | | 160-169 | | | 170-179 | | | >=180 | |
|  | |  | | | |  | | |  | |  | |  | | |  | | |  | |
| **Number of standard dose drugs** | | 0.5 | | | | 1.0 | | | 2.0 | |  | | 3.0 | | | 3.5 | | | 4.0 | |
| **35-44 years** | |  | | | |  | | |  | |  | |  | | |  | | |  | |
| **percent of all untreated HT** | | 40.0% | | | | 12.8% | | | 24.5% | |  | | 14.4% | | | 3.6% | | | 4.7% | |
| **percent of stage** | | 51.8% | | | | 16.5% | | | 31.7% | |  | | 63.6% | | | 15.8% | | | 20.7% | |
| **BP change after treatment** | | 4.2 | | | | 8.4 | | | 18.2 | |  | | 27.6 | | | 36.6 | | | 42.0 | |
| **RR CHD after treatment** | | 0.89 | | | | 0.80 | | | 0.61 | |  | | 0.47 | | | 0.37 | | | 0.32 | |
| **RR stroke after treatment** | | 0.86 | | | | 0.74 | | | 0.52 | |  | | 0.37 | | | 0.27 | | | 0.22 | |
| **Annual anti-HT medication cost** | | ¥ 33.68 | | | | ¥ 67.36 | | | ¥ 124.13 | |  | | ¥ 184.52 | | | ¥ 246.60 | | | ¥ 269.43 | |
|  | |  | | | |  | | |  | |  | |  | | |  | | |  | |
| **45-54 years** | |  | | | |  | | |  | |  | |  | | |  | | |  | |
| **percent of all untreated HT** | | 25.1% | | | | 16.0% | | | 26.5% | |  | | 14.6% | | | 7.5% | | | 10.3% | |
| **percent of stage** | | 37.1% | | | | 23.7% | | | 39.2% | |  | | 45.1% | | | 23.0% | | | 31.9% | |
| **BP change after treatment** | | 4.2 | | | | 8.4 | | | 18.2 | |  | | 27.6 | | | 36.6 | | | 42.0 | |
| **RR CHD after treatment** | | 0.90 | | | | 0.82 | | | 0.65 | |  | | 0.52 | | | 0.42 | | | 0.37 | |
| **RR stroke after treatment** | | 0.88 | | | | 0.77 | | | 0.57 | |  | | 0.42 | | | 0.32 | | | 0.27 | |
| **Annual anti-HT medication cost** | | ¥ 33.68 | | | | ¥ 67.36 | | | ¥ 124.13 | |  | | ¥ 184.52 | | | ¥ 246.60 | | | ¥ 269.43 | |
|  | |  | | | |  | | |  | |  | |  | | |  | | |  | |
| **55-64 years** | |  | | | |  | | |  | |  | |  | | |  | | |  | |
| **percent of all untreated HT** | | 22.6% | | | | 14.9% | | | 21.6% | |  | | 19.3% | | | 8.8% | | | 12.7% | |
| **percent of stage** | | 38.2% | | | | 25.2% | | | 36.6% | |  | | 47.3% | | | 21.5% | | | 31.2% | |
| **BP change after treatment** | | 4.2 | | | | 8.4 | | | 18.2 | |  | | 27.6 | | | 36.6 | | | 42.0 | |
| **RR CHD after treatment** | | 0.91 | | | | 0.83 | | | 0.68 | |  | | 0.55 | | | 0.45 | | | 0.41 | |
| **RR stroke after treatment** | | 0.89 | | | | 0.79 | | | 0.60 | |  | | 0.46 | | | 0.36 | | | 0.31 | |
| **Annual anti-HT medication cost** | | ¥ 33.68 | | | | ¥ 67.36 | | | ¥ 124.13 | |  | | ¥ 184.52 | | | ¥ 246.60 | | | ¥ 269.43 | |
|  | |  | | | |  | | |  | |  | |  | | |  | | |  | |
| **65-74 years** | |  | | | |  | | |  | |  | |  | | |  | | |  | |
| **percent of all untreated HT** | | 18.5% | | | | 16.5% | | | 30.0% | |  | | 19.1% | | | 6.0% | | | 9.8% | |
| **percent of stage** | | 0.0% | | | | 0.0% | | | 46.2% | |  | | 54.6% | | | 17.3% | | | 28.1% | |
| **BP change after treatment** | | 0.0 | | | | 0.0 | | | 8.4 | |  | | 18.2 | | | 27.6 | | | 36.6 | |
| **RR CHD after treatment** | | 1.00 | | | | 1.00 | | | 0.86 | |  | | 0.72 | | | 0.61 | | | 0.52 | |
| **RR stroke after treatment** | | 1.00 | | | | 1.00 | | | 0.82 | |  | | 0.65 | | | 0.52 | | | 0.42 | |
| **Annual anti-HT medication cost** | | ¥ - | | | | ¥ - | | | ¥ 67.36 | |  | | ¥ 124.13 | | | ¥ 184.52 | | | ¥ 246.60 | |
|  | |  | | | |  | | |  | |  | |  | | |  | | |  | |
| **75-84 years** | |  | | | |  | | |  | |  | |  | | |  | | |  | |
| **percent of all untreated HT** | | 18.3% | | | | 12.6% | | | 26.6% | |  | | 16.5% | | | 12.9% | | | 13.1% | |
| **percent of stage** | | 0.0% | | | | 0.0% | | | 46.3% | |  | | 38.8% | | | 30.4% | | | 30.8% | |
| **BP change after treatment** | | 0.0 | | | | 0.0 | | | 8.4 | |  | | 18.2 | | | 27.6 | | | 36.6 | |
| **RR CHD after treatment** | | 1.00 | | | | 1.00 | | | 0.90 | |  | | 0.79 | | | 0.70 | | | 0.62 | |
| **RR stroke after treatment** | | 1.00 | | | | 1.00 | | | 0.89 | |  | | 0.77 | | | 0.67 | | | 0.59 | |
| **Annual anti-HT medication cost** | | ¥ - | | | | ¥ - | | | ¥ 67.36 | |  | | ¥ 124.13 | | | ¥ 184.52 | | | ¥ 246.60 | |
|  | |  | | | |  | | |  | |  | |  | | |  | | |  | |

**S1 Table 6. Distributions of main input parameters used in probabilistic sensitivity analyses**

| **Parameter** | **Distribution sampled** | **Main estimate** | **Measure of dispersion** | **Magnitude of dispersion** | **Sources for estimates** |
| --- | --- | --- | --- | --- | --- |
| **Systolic Hypertension** | Normal | Percent of population | Standard error (%) |  |  |
| **Stage 1 (140-159 mmHg)**  Men  35-44 years  45-54  55-64  65-74  75-84 |  | 7.1  13.8  21.7  25.6  25.6* |  | 0.6  1.0  1.4  2.0  2.0* | InterASIA Study[^20^](#_ENREF_20) |
| Women  35-44 years  45-54  55-64  65-74  75-84 |  | 4.8  13.3  19.4  28.5  28.5* |  | 0.5  1.0  1.3  2.1  2.1* |  |
| **Stage 2 (140-159 mmHg)**  Men  35-44 years  45-54  55-64  65-74  75-84 |  | 1.1  4.1  11.8  16.1  16.1* |  | 0.2  0.6  1.0  1.8  1.8* | InterASIA Study[^20^](#_ENREF_20) |
| Women  35-44 years  45-54  55-64  65-74  75-84 |  | 1.4  6.4  13.4  15.4  15.4* |  | 0.2  0.7  1.1  1.6  1.6* |  |
| **Stage 1 (140-159 mmHg)**  Men  35-44 years  45-54  55-64  65-74  75-84 | Normal | Mean (mmHg)  146.5  146.9  148.0  148.1  148.1* | Standard error (mmHg) | 0.5  0.5  0.4  0.5  0.5* | InterASIA Study[^20^](#_ENREF_20) |
| Women  35-44 years  45-54  55-64  65-74  75-84 |  | 146.1  148.1  147.4  149.4  149.4* |  | 0.6  1.5  1.1  1.3  1.3* |  |
| **Stage 2 (140-159 mmHg)**  Men  35-44 years  45-54  55-64  65-74  75-84 |  | 173.9  174.6  176.9  179.7  179.7* |  | 2.8  1.8  1.8  2.2  2.2* | InterASIA Study[^20^](#_ENREF_20) |
| Women  35-44 years  45-54  55-64  65-74  75-84 |  | 173.2  175.4  174.3  172.3  172.3* |  | 2.1  1.5  1.1  1.6  1.6* |  |
| **Hypertension treatment**  Men  35-44 years  45-54  55-64  65-74  75-84 | Normal | Percent of whole population  2.4  6.6  11.0  14.4  14.4 | Standard error (%) | 0.4  0.7  1.0  1.6  1.6 | InterASIA Study[^20^](#_ENREF_20) |
| Women  35-44 years  45-54  55-64  65-74  75-84 |  | 2.3  10.1  14.4  16.7  16.7* |  | 0.3  0.8  1.1  1.7  1.7* |  |
| **Case-fatality** | Normal | Percent of cases dying of any cause | 95% confidence interval |  |  |
| **Hospitalized acute myocardial Infarction**  Men  35-44 years  45-54  55-64  65-74  75-84 |  | 5.0  15.0  18.5  25.0  50.0 |  | 0.4  1.1  1.3  1.8  3.5 | Bridging the Gap in Acute Coronary Syndromes Study (Multi-provincial hospital based study)[^16^](#_ENREF_16)^,^ [^17^](#_ENREF_17) |
| Women  35-44 years  45-54  55-64  65-74  75-84 |  | 15.0  20.0  20.0  30.0  50.0 |  | 1.1  1.4  1.4  2.1  3.5 |  |
| **Acute stroke**  Men  35-44 years  45-54  55-64  65-74  75-84 | Normal | 25.0  18.0  12.0  20.0  45.0 |  | 0.4  0.4  0.4  0.4  0.4 | Sino-MONICA Beijing surveillance study[^18^](#_ENREF_18) |
| Women  35-44 years  45-54  55-64  65-74  75-84 |  | 18.0  14.0  15.0  20.0  45.0 |  | 0.6  0.6  0.6  0.6  0.6 |  |
| **Hospitalized acute CVD costs**  Hospitalized Stroke  Angina pectoris  Acute MI  Acute MI with percutaneous coronary intervention  Acute MI with coronary artery bypass graft surgery  Acute heart failure | Normal | 2010 Chinese RMB  8,015  7,887  15,774  39,434  80,671  8,885 | Standard deviation | 2,706  2,706  2,706  2,706  2,706  2,706 | National variation in hospital length of stay,  China PEACE Study[^19^](#_ENREF_19) |
| **Chronic CVD costs**  First year after MI  Subsequent years after MI  First year after stroke  Subsequent years after stroke | Normal | 2010 Chinese RMB  3,245  2,261  1,980  1,275 | 95% confidence interval | 67  47  41  26 | Variation in outpatient visit costs between rural and urban China, World Health Organization CHOICE regression analysis[^21^](#_ENREF_21) |
| **Blood pressure change** | Normal | See manuscript table 2 and Appendix table 10 | Standard deviation | 0.15 | Law and Wald trials meta-analysis (2003)[^22^](#_ENREF_22) |
| **Relative risk of CVD**  **Coronary heart disease**  Men  35-44 years  45-54  55-64  65-74  75-84 | Normal | Beta coefficients; Per 1 mmHg SBP or 0.5 mmHg DBP  0.0325  0.0310  0.0300  0.0265  0.0230 | 95% confidence interval | Per 1 mmHg SBP  or 0.5 mmHg DBP  0.00352  0.00163  0.00124  0.00174  0.00174 | Law, Morris, and Wald trials meta-analysis and Prospective Studies Collaboration  (2009)[^13^](#_ENREF_13)^,^ [^23^](#_ENREF_23) |
| Women  35-44 years  45-54  55-64  65-74  75-84 |  | 0.0320  0.0320  0.0295  0.0245  0.0228 |  | 0.00348  0.00144  0.00124  0.00165  0.00165 |  |
| **Stroke**  Men  35-44 years  45-54  55-64  65-74  75-84 |  | 0.0500  0.0460  0.0420  0.0370  0.0230 |  | 0.00472  0.00343  0.00214  0.00401  0.00218 |  |
| Women  35-44 years  45-54  55-64  65-74  75-84 |  | 0.0470  0.0450  0.0414  0.0345  0.0267 |  | 0.00476  0.00318  0.00220  0.00459  0.00208 |  |
| **Bundled treatment costs (medications, monitoring, and side effects)** | Normal | See manuscript table 2 | Coefficient of variation | 0.05 | Distribution of bundled costs when component costs were assembled (driven by medication costs; see Appendix C) |
| **Quality of life penalty** | Normal | See manuscript table 2 | Standard deviation | 0.258 | Side effect frequency in Law and Wald trials meta-analysis (2003)[^22^](#_ENREF_22) |

**S1Table 7. Cost effectiveness hypertension control in Chinese adults with and without adding separate components of program costs. All estimates are incremental cost-effectiveness ratios (ICERs), compared with the prior strategy. Results are in 2015 international dollars (2015 Chinese RMB). All results reported as cost-saving describe strategies projected to be less costly and more effective than the prior strategy.**

| Strategy | **Strategy 1:** Treat all stage two hypertension patients to goal of <140/90 mmHg if age 35-64 years, goal of 150/90 mmHg if age ≥65 in addition to CVD patients | **Strategy 2:** Treat stage two and stage one, goal <140/90 mmHg if age 35-64 years, goal of 150/90 mmHg if age ≥65, in addition to CVD patients |
| --- | --- | --- |
| Comparator for ICER | Treat only CVD patients (base case) | Strategy 1 |
| Hypertension treatment alone (costs of medications, monitoring, side effects only) | Cost-saving | $12,000 (¥ 42,200)§ |
| Add cost of systematic hypertension screening program in ages 35-84 years¶ | $8,200 (¥ 28,700)∆ | $12,000 (¥ 42,200)§ |
| Cost of implementing Essential Medicines use  Add 5% of drug expenditures | Cost-saving | $12,200 (¥ 43,000)§ |
| Add 15% of drug expenditures | $100 (¥ 200)∆ | $12,600 (¥ 44,500)§ |
| Cost of administering hypertension control program  Add 5% of total screening and treatment costs for program administration | $400 (¥ 1,500)∆ | $12,800 (¥ 45,000)§ |
| Include all added program costs (main analysis)  Add screening program, 15% of drug expenditures, and 5% program administration cost | $8,900 (¥ 31,300)∆ | $13,500 (¥ 47,400)§ |

¶Normotensive once every two years, prehypertensive once yearly

∆Less than 1 x China’s gross domestic product per capita

§Less than 2 x China’s gross domestic product per capita, but not less than 1 x GDP per capita

**Meta-analysis of East Asian BP treatment trials**

Because the PROGRESS trial results suggested that BP lowering for stroke prevention was more effective in East Asian participants, we performed our own meta-analysis of BP treatment trials in East Asian participants in order to see if the effectiveness of BP treatment differed between East Asian and European descent participants. Four East Asian population trials used in the Law, Morris, and Wald antihypertensive medication trials meta-analysis were reviewed,[^24-27^](#_ENREF_24) and a major trial published since the Law, Morris and Wald meta-analysis was also reviewed. Only three placebo-controlled treatment trials were used in our meta-analysis of stroke prevention effectiveness.[^26-28^](#_ENREF_26) The result of this meta-analysis was that the estimates were homogeneous and the pooled relative risk estimate for a systolic BP reduction of 10 mm Hg [0.59 (0.49—0.71)] was almost identical to the overall Law, Morris and Wald estimate used for this analysis [0.59 (0.52—0.67)]. Only two trials reported CHD outcomes;[^26^](#_ENREF_26)^,^ [^27^](#_ENREF_27) the relative risk estimates with a 10 mm Hg reduction in systolic BP were considered too heterogeneous and uncertain to include as an alternate relative risk estimate for East Asian patients.

**Stroke** Meta-analysis (exponential form)

| Pooled 95% CI Asymptotic No. of

Method | Est Lower Upper z_value p_value studies

-------+----------------------------------------------------

Fixed | 0.586 0.485 0.708 -5.529 0.000 3

Random | 0.586 0.485 0.708 -5.529 0.000

Test for heterogeneity: Q= 1.081 on 2 degrees of freedom (p= 0.583)

**CHD** Meta-analysis (exponential form)

| Pooled 95% CI Asymptotic No. of

Method | Est Lower Upper z_value p_value studies

-------+----------------------------------------------------

Fixed | 0.583 0.322 1.058 -1.775 0.076 2

Random | 0.625 0.239 1.631 -0.961 0.337

Test for heterogeneity: Q= 2.479 on 1 degrees of freedom (p= 0.115)

**Appendix C. Estimation of median and upper lower range of costs of four antihypertensive medications costs in the national, Shanghai municipality, and Yunnan province essential medication lists.**

We explored the profile of antihypertensive drugs prices under the essential medicine system initiated in 2009 by China’s national government. We used representative and publicly accessible essential drug lists from the central government (<http://www.sdpc.gov.cn/zcfb/zcfbtz/2009tz/t20091002_305945.htm>), Shanghai municipality (<http://www.shdrc.gov.cn/searchresult_detail.jsp?main_artid=18980>; higher cost) and Yunnan province (<http://xxgk.yn.gov.cn/canton_model13/newsview.aspx?id=715703>; lower cost). Compared indicators were drugs number, median, maximum, minimum, quartile and 95%CI of annual cost analyzed with SPSS 16.0 and Excel 2007 software. First, all injected medications were removed from the data; second, short-acting nifedipine and other short-acting calcium-channel blockers were removed from the data (controlled release and sustained release formulations were retained). All drugs had to be earmarked for use in primary health centers. The four drug classes of interest for this analysis were the most standard, lowest cost, antihypertensive agents: beta blockers (β-B), angiotensin converting enzyme inhibitors (ACE I), calcium channel blockers (CCB), and thiazide diuretics. For CCBs, when two lower priced agents were available, (e.g., nitrendipine and nimodipine in Shanghai) we used the median between these prices. For the national and Yunnan lists, only the lowest priced CCB was used. We only used hydrochlorothiazide for the thiazide diuretic. Since no thiazide diuretic was on the Yunnan or Shanghai lists, we substituted the national list price when calculating average prices. Annual cost was calculated according to one standard daily dose, or one-half standard dose according to by the Law, Morris and Wald definition used in this analysis. In cases where the half-standard dose price was higher than the full-standard dose price, the lower price was assumed, regardless of one or one-half dose. Daily costs were inflated to annual costs by multiplying by 365.

Table1-1 National Antihypertensive drugs annual cost (CHY)*—one full of standard dose

| drugs classes | N | median | highest | lowest | Quartile | | 95%CI for median | |
| --- | --- | --- | --- | --- | --- | --- | --- | --- |
|  |  |  |  |  | Up | Low | Upper | lower |
| β-B | 28 | 85.41 | 715.40 | 38.54 | 85.41 | 65.70 | 431.31 | 211.18 |
| ACEI | 27 | 109.50 | 782.72 | 85.41 | 109.50 | 91.25 | 437.34 | 232.57 |
| CCB: nifedipine | 8 | 56.45 | 1153.40 | 31.97 | 685.14 | 43.38 | 674.65 | -88.47 |
| Thiazide† | 3 | 17.34 | 18.07 | 14.24 | 17.34 | 14.24 | 21.60 | 11.49 |

Table1-2 National Antihypertensive drugs annual cost (CHY)*—one half full of standard dose

| drugs classes | N | median | highest | lowest | Quartile | | 95%CI for median | |
| --- | --- | --- | --- | --- | --- | --- | --- | --- |
|  |  |  |  |  | Up | Low | Upper | lower |
| β-B | 28 | 42.70 | 357.70 | 19.27 | 305.69 | 32.85 | 215.66 | 105.59 |
| ACEI | 27 | 54.75 | 391.36 | 42.71 | 302.27 | 45.62 | 218.67 | 116.29 |
| CCB: nifedipine | 8 | 28.22 | 576.70 | 15.99 | 342.57 | 21.69 | 337.32 | -44.23 |
| Thiazide† | 3 | 8.66 | 9.03 | 7.12 | / | 7.11 | 10.80 | 5.74 |

Table 2-1 Shanghai Antihypertensive drugs annual cost (CHY)*--one full standard dose

| drugs classes | N | median | highest | lowest | Quartile | | 95%CI for median | |
| --- | --- | --- | --- | --- | --- | --- | --- | --- |
|  |  |  |  |  | Up | Low | Upper | lower |
| β-B | 17 | 1003.80 | 2978.40 | 259.15 | 1297.50 | 419.75 | 1419.10 | 621.59 |
| ACEI | 26 | 586.43 | 2654.07 | 13.87 | 1359.60 | 171.85 | 1062.10 | 512.03 |
| CCB: nifedipine | 5 | 179.58 | 521.22 | 43.80 | 1808.80 | 301.73 | 1809.70 | 494.73 |
| CCB: nitrendipine | 3 | 23.12 | 672.21 | 7.30 | / | 7.30 | 1176.70 | -708.28 |
| Thiazide† | 0 | 0 | 0 | 0 | 0 | 0 | 0 | 0 |

Table 2-2 Shanghai Antihypertensive drugs annual cost (CHY)*--one half full standard dose

| drugs classes | N | median | highest | lowest | Quartile | | 95%CI for median | |
| --- | --- | --- | --- | --- | --- | --- | --- | --- |
|  |  |  |  |  | Up | Low | Upper | lower |
| β-B | 17 | 501.88 | 1489.20 | 129.58 | 648.74 | 209.88 | 709.55 | 310.80 |
| ACEI | 26 | 293.22 | 1327.04 | 6.94 | 679.81 | 85.92 | 531.07 | 256.01 |
| CCB: nifedipine | 9 | 758.39 | 1230.57 | 100.37 | 904.39 | 150.87 | 904.87 | 247.36 |
| CCB: nitrendipine | 3 | 11.55 | 336.10 | 3.65 | / | 3.65 | 588.35 | -354.14 |
| Thiazide† | 0 | 0 | 0 | 0 | 0 | 0 | 0 | 0 |

Table 3-1 Yunnan Antihypertensive drugs annual cost (CHY)*--one full standard dose

| drugs classes | N | median | highest | lowest | Quartile | | 95%CI for median | |
| --- | --- | --- | --- | --- | --- | --- | --- | --- |
|  |  |  |  |  | Up | Low | Upper | lower |
| β-B | 3 | 31.39 | 33.58 | 26.36 | / | 26.36 | 39.63 | 21.24 |
| ACEI | 4 | 22.99 | 29.20 | 17.52 | 28.83 | 17.70 | 32.95 | 13.39 |
| CCB: nitrendipine | 3 | 8.03 | 939.88 | 6.57 | / | 6.57 | 1655.70 | -1019.40 |
| Thiazide† | 0 | 0 | 0 | 0 | 0 | 0 | 0 | 0 |

Table 3-2 Yunnan Antihypertensive drugs annual cost (CHY)*--one half full standard dose

| drugs classes | N | median | highest | lowest | Quartile | | 95%CI for median | |
| --- | --- | --- | --- | --- | --- | --- | --- | --- |
|  |  |  |  |  | Up | Low | Upper | lower |
| β-B | 3 | 15.69 | 16.79 | 13.18 | / | 13.18 | 19.81 | 10.62 |
| ACEI | 4 | 11.49 | 14.60 | 8.76 | 14.41 | 8.85 | 16.47 | 6.69 |
| CCB: nitrendipine | 3 | 4.0150 | 469.94 | 3.28 | / | 3.28 | 827.84 | -509.68 |
| Thiazide† | 0 | 0 | 0 | 0 | 0 | 0 | 0 | 0 |

*2009 price in Chinese RMB listed on government websites

†only includes hydrochlorothiazide

**The figures below show the medians and interquartile ranges of annual drug costs for the national, Shanghai, and Yunnan essential medicines lists for the four antihypertensive drug classes.**

ACEI annual cost comparison among national, Shanghai, and Yunnan


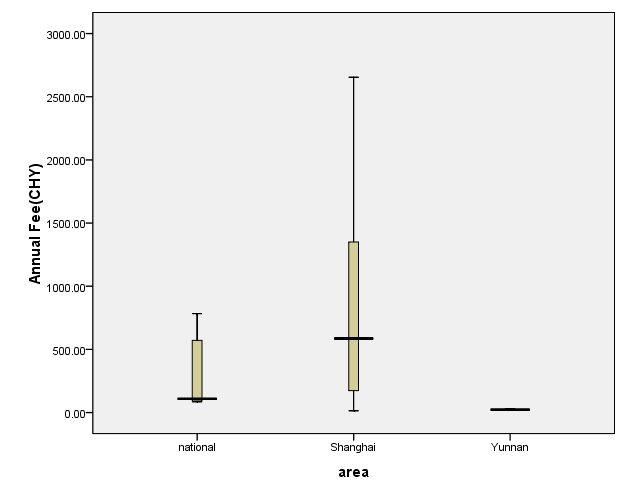


CCB annual cost comparison among national, Shanghai, and Yunnan
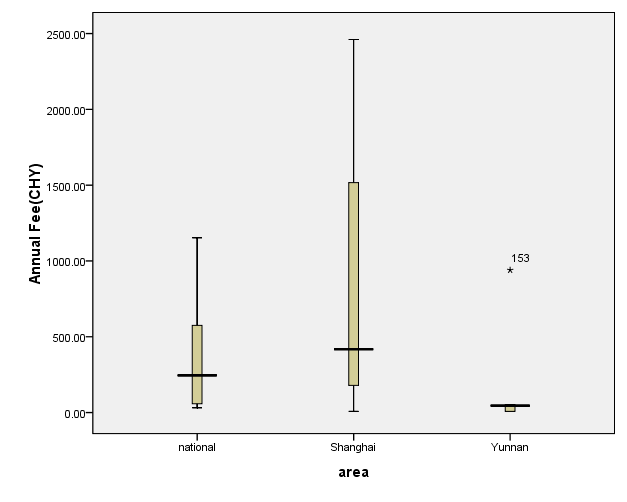


Thiazide annual cost comparison among national, Shanghai, and Yunnan

Hydrochlorothiazide only available on the national drug list


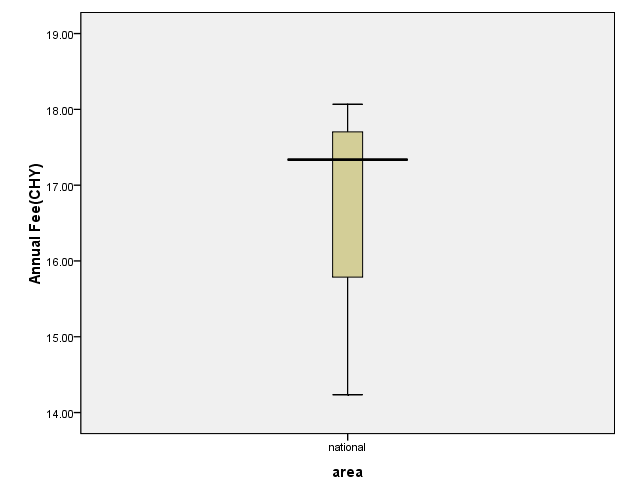


β-blocker annual cost comparison among national, Shanghai, and Yunnan


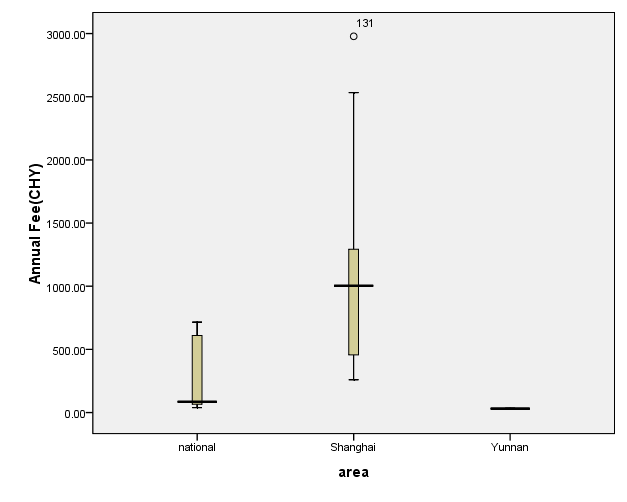


**Appendix References:**

1. Moran A, Zhao D, Gu D, et al. The future impact of population growth and aging on coronary heart disease in China: projections from the Coronary Heart Disease Policy Model-China. *BMC Public Health* 2008; **8**: 394.

2. He J, Neal B, Gu D, et al. International collaborative study of cardiovascular disease in Asia: design, rationale, and preliminary results. *Ethn Dis* 2004; **14**(2): 260-8.

3. Zhao D, Liu J, Wang W, et al. Epidemiological transition of stroke in China: twenty-one-year observational study from the Sino-MONICA-Beijing Project. *Stroke; a journal of cerebral circulation* 2008; **39**(6): 1668-74.

4. Gu D, Kelly TN, Wu X, et al. Blood pressure and risk of cardiovascular disease in Chinese men and women. *American journal of hypertension* 2008; **21**(3): 265-72.

5. He J, Gu D, Wu X, et al. Major causes of death among men and women in China. *The New England journal of medicine* 2005; **353**(11): 1124-34.

6. Lozano R, Murray C.J.L., Lopez, A.D., and Satoh, T. Miscoding and misclassification of ischaemic heart disease mortality. Global Programme on Evidence for Health Policy Working Paper No. 12. . *Geneva, World Health Organization* 2001.

7. Liu J, Hong Y, D'Agostino RB, Sr., et al. Predictive value for the Chinese population of the Framingham CHD risk assessment tool compared with the Chinese Multi-Provincial Cohort Study. *JAMA* 2004; **291**(21): 2591-9.

8. Keil U, Kuulasmaa K. WHO MONICA Project: risk factors. *International journal of epidemiology* 1989; **18**(3 Suppl 1): S46-55.

9. Moran AE, Forouzanfar MH, Roth G, et al. The Global Burden of Ischemic Heart Disease in 1990 and 2010: The Global Burden of Disease 2010 Study. *Circulation* 2014.

10. Salomon JA, Vos T, Hogan DR, et al. Common values in assessing health outcomes from disease and injury: disability weights measurement study for the Global Burden of Disease Study 2010. *Lancet* 2012; **380**(9859): 2129-43.

11. Ministry of Health. China health statistics yearbook in 2011. Beijing: China Union Medical University Press; 2011.

12. Huffman MD, Rao KD, Pichon-Riviere A, et al. A cross-sectional study of the microeconomic impact of cardiovascular disease hospitalization in four low- and middle-income countries. *PloS one* 2011; **6**(6): e20821.

13. Law MR, Morris JK, Wald NJ. Use of blood pressure lowering drugs in the prevention of cardiovascular disease: meta-analysis of 147 randomised trials in the context of expectations from prospective epidemiological studies. *BMJ* 2009; **338**: b1665.

14. When the beat is off--atrial fibrillation. April 30, 2014. <http://www.strokeassociation.org/STROKEORG/LifeAfterStroke/HealthyLivingAfterStroke/UnderstandingRiskyConditions/When-the-Beat-is-Off---Atrial-Fibrillation_UCM_310782_Article.jsp> (accessed August 29 2014).

15. Gu D, Reynolds K, Wu X, et al. Prevalence, awareness, treatment, and control of hypertension in china. *Hypertension* 2002; **40**(6): 920-7.

16. Niu S, Zhao D, Zhu J, et al. The association between socioeconomic status of high-risk patients with coronary heart disease and the treatment rates of evidence-based medicine for coronary heart disease secondary prevention in China: Results from the Bridging the Gap on CHD Secondary Prevention in China (BRIG) Project. *Am Heart J* 2009; **157**(4): 709-15 e1.

17. Wang M, Moran AE, Liu J, et al. Cost-effectiveness of optimal use of acute myocardial infarction treatments and impact on coronary heart disease mortality in China. *Circ Cardiovasc Qual Outcomes* 2014; **7**(1): 78-85.

18. Zhao D, Liu J, Wang W, et al. Epidemiological transition of stroke in China: twenty-one-year observational study from the Sino-MONICA-Beijing Project. *Stroke* 2008; **39**(6): 1668-74.

19. Li Q, Lin Z, Masoudi FA, et al. National trends in hospital length of stay for acute myocardial infarction in China. *BMC cardiovascular disorders* 2015; **15**: 9.

20. Gu D, Reynolds K, Wu X, et al. Prevalence, awareness, treatment, and control of hypertension in china. *Hypertension* 2002; **40**(6): 920-7.

21. Country-specific unit costs: World Health Organization-CHOosing Interventions that are Cost Effective (WHO-CHOICE). 2011. <http://www.who.int/choice/country/country_specific/en/> (accessed March 27 2015).

22. Law MR, Wald NJ, Morris JK, Jordan RE. Value of low dose combination treatment with blood pressure lowering drugs: analysis of 354 randomised trials. *BMJ* 2003; **326**(7404): 1427.

23. Moran AE, Odden MC, Thanataveerat A, et al. Cost-effectiveness of hypertension therapy according to 2014 guidelines. *The New England journal of medicine* 2015; **372**(5): 447-55.

24. Comparison of the effects of beta blockers and calcium antagonists on cardiovascular events after acute myocardial infarction in Japanese subjects. *Am J Cardiol* 2004; **93**(8): 969-73.

25. Ueshima K, Fukami K, Hiramori K, et al. Is angiotensin-converting enzyme inhibitor useful in a Japanese population for secondary prevention after acute myocardial infarction? A final report of the Japanese Acute Myocardial Infarction Prospective (JAMP) study. *Am Heart J* 2004; **148**(2): e8.

26. Liu L, Zhang Y, Liu G, Li W, Zhang X, Zanchetti A. The Felodipine Event Reduction (FEVER) Study: a randomized long-term placebo-controlled trial in Chinese hypertensive patients. *J Hypertens* 2005; **23**(12): 2157-72.

27. Liu L, Wang JG, Gong L, Liu G, Staessen JA. Comparison of active treatment and placebo in older Chinese patients with isolated systolic hypertension. Systolic Hypertension in China (Syst-China) Collaborative Group. *J Hypertens* 1998; **16**(12 Pt 1): 1823-9.

28. Rodgers A, Chapman N, Woodward M, et al. Perindopril-based blood pressure lowering in individuals with cerebrovascular disease: consistency of benefits by age, sex and region. *J Hypertens* 2004; **22**(3): 653-9.

**S1 Fig. Results of probabilistic sensitivity analyses of treating all only stage two hypertension (blue dots) and treating all untreated stage one and stage two hypertension (red dots). Both strategies are compared with a base case in which hypertension was treated in all patients with pre-existing cardiovascular disease.**


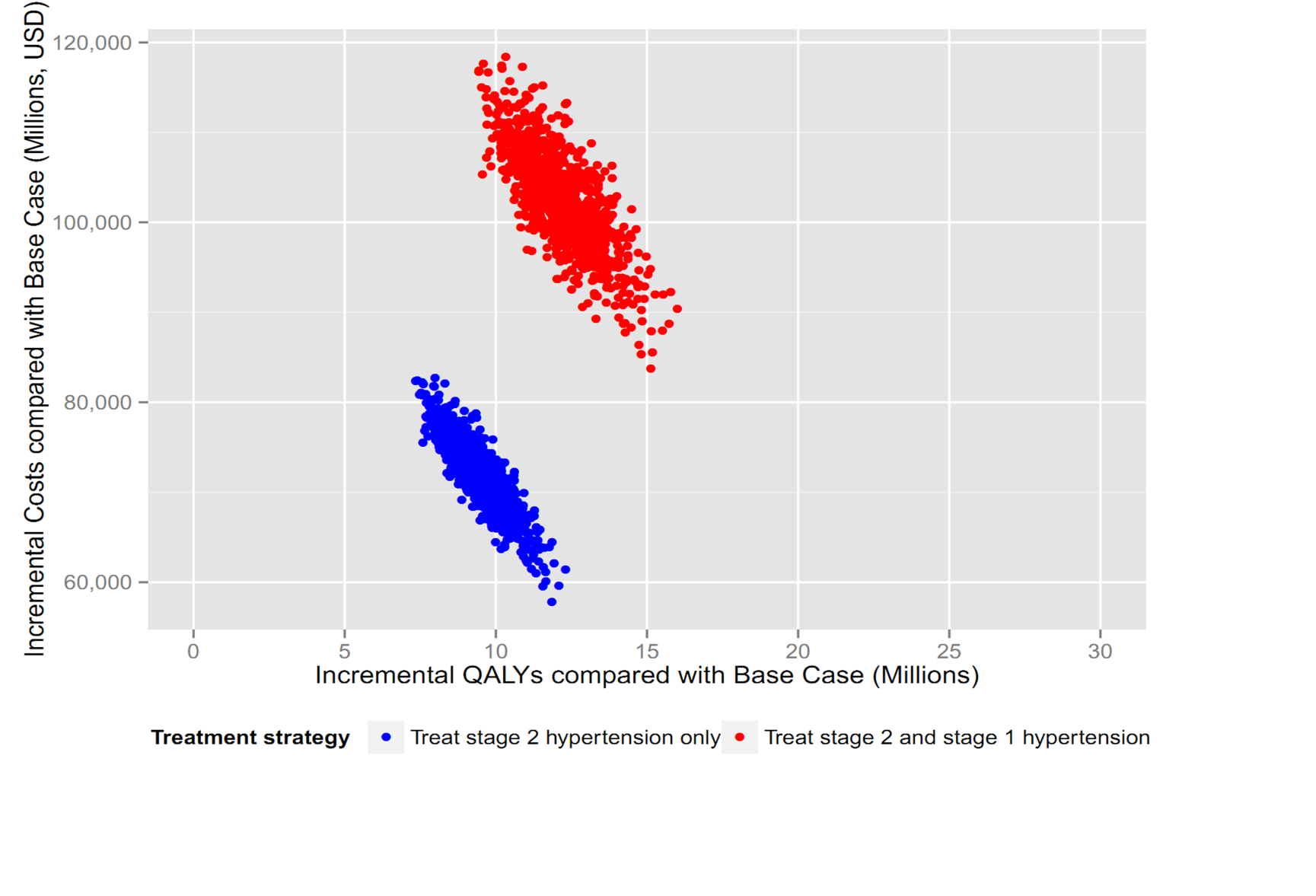

Supplement: S1 Text — (DOCX) [file pmed.1001860.s010.docx]
