# Supplementary material for: The Cost-Effectiveness of Low-Cost Essential Antihypertensive Medicines for Hypertension Control in China: A Modelling Study
Source: PLoS Med. 2015 Aug 4;12(8):e1001860. doi: 10.1371/journal.pmed.1001860 (PMC4524696; doi:10.1371/journal.pmed.1001860)
Supplement: S1 User Agreement — (DOC) [file pmed.1001860.s011.doc]

# Cardiovascular Disease Policy Model Software Commons

Developer Project Participation Agreement

Project: Cardiovascular Disease Policy Model Software Commons (the “Commons”)

Participant Name: Institution: ____________________________________

To participate in the Commons, I acknowledge and agree to the following:

The Commons I understand that the Commons has been established to provide a research environment of collaborative development for software and related documentation stemming from the Cardiovascular Disease Policy Model. In order to disseminate these new research tools effectively to academic and commercial users, I recognize that it is important to manage the copyrights and other intellectual property rights arising from all contributions to the Commons, including my own. Accordingly, I agree to adhere to the following Project terms.

The Commons

Property “Project Property” means software code and related documentation authored or developed by me, either solely or jointly with others, that (i) is developed within or for the Commons, and (ii) is contributed to the Commons. “Project Property” also includes any and all proprietary rights arising from such software code inputs, and related documentation, including copyrights and other proprietary rights.

Reporting

Contributions I will participate in the contribution tracking mechanisms provided by the Commons. I understand that these mechanisms will require me to contribute software code and inputs developed as part of the Commons to a designated repository/server using protocols established by the Commons. These protocols may be updated from time to time.

Use of Project

Property I understand that while my institution is a participating institution in the Commons and this Participation Agreement is in effect, I may work with, modify, and conduct research with all software within the Commons. I acknowledge, however, that I may not transfer, assign, or license rights in any Project Property (other than transferring or assigning rights to my institution) unless I first obtain the authorization of the lead institution of the Commons, currently Columbia University. I agree not to disclose or publish any code or information that others have developed within or for the Commons and that is not yet publicly available, unless I am specifically authorized to do so by the Commons Director or Commons Executive Committee. I agree not to involve others in Commons work unless they have signed a Commons Participation Agreement or are approved as outside consultants or collaborators by the Commons Director or Executive Committee. I understand this applies to others in the laboratory in which I work.

Outside Property Prior to integrating external code, inputs, or libraries into work that is expected to become Project Property (i.e., be contributed to the Commons), I will review the specific academic license or terms of use to confirm that there are not restrictions on use or distribution of this external code or inputs by academic or commercial users and that the terms are consistent with the Commons distribution program. If I am unsure what the terms of use are or what they mean, I will contact my institution’s technology transfer office or the Commons Executive Committee member at my institution.

I will not contribute to the Commons or use in Project Property:

- any proprietary information of any prior employer or any third party, including but not limited to confidential information or software code, if I know or have reason to know that my disclosure and/or use of the proprietary information in Commons work or the public availability of Commons materials is inconsistent with my obligations to the prior employer or with my or my institution’s obligations to any third party.
- any proprietary subject matter in which I assert a personal claim inconsistent with my participation in the Project on the terms described in this Participation Agreement or my institution’s obligations to any third party.

Assignment I will assign to my institution upon request any and all right, title, and interest I may have in and to Project Property, and will execute all necessary documents and otherwise provide reasonable assistance, during and subsequent to my affiliation with my institution, to enable my institution to obtain, maintain, deploy, and enforce for itself and its nominees, agents, and licensees, any and all patents, copyrights, trademarks, and other legal means of ownership and protection for Project Property.

Copyright Software code, inputs, and related documentation I author or co-author within or for the Project may be works made for hire and ownership shall vest with my institution. If any such works cannot be deemed to be works made for hire, I hereby assign right, title, and interest in and to such works to my institution. I agree to sign assignments of my right, title, and interest in and to such works to my institution, if my institution so requests. However, I am not required by this Participation Agreement to assign to my institution the copyright to books or articles I may write, alone or with others, and submit to a scientific press or journal for publication.

Permission For works and rights I own personally, but which I voluntarily provide to, or include in, work for the Commons, I hereby grant to the Commons a paid-up, royalty-free, non-exclusive, irrevocable, worldwide right to use such works and rights in any manner, including but not limited to creation of derivative works, reproduction of such work and derivative works in copies, distribution of such work and derivative works, public display and performance of such work and derivative works, and sale and licensing of such work and derivative works to others as part of Project-related materials.

Revenue I acknowledge that revenue received through software licensing will be used by the Commons for Cost Recovery (see below), until a certain amount has been received. This ceiling amount will be established by the Commons Director on an annual basis. Once the ceiling is surpassed, the revenue will be shared among the participating institutions, the research sponsors and equally among all active Project Participants. I acknowledge that there is no obligation on the part of any institution or person involved with the Commons to maximize my share of revenue, to seek recovery of revenue in the event of default of any third party, or otherwise to act responsive to my personal interest in any such revenue. I shall assert no claim to revenue due or received or designated as research or other non-royalty funding.

Cost Recovery I agree that revenue related to licensing of the Commons software may be used for the benefit of the Commons, including software development services, intellectual property expenses, and other such expenses relating to the development of software and research tools in the Commons which have been approved by the Commons Director.

Dispute I agree to make good faith efforts to resolve any concerns I may have regarding this Agreement or the Project through discussion with the Commons Director and Executive Committee and my institution’s technology transfer office.

Succession I agree that discharge of these obligations with regard to the Project will be an obligation of my executors, administrators, or other legal representatives or assignees.

Other Obligations I understand that this Participation Agreement does not fulfill, waive, replace, or relieve me from reporting obligations and otherwise complying with my institution’s policies, state law, and Federal law and agency regulations pertaining to ethics in public service and conflict of interest. I further understand and agree that as a Participant in the Project, I am bound by any obligations the Commons may have to third parties with respect to software and documentation that has been contributed to the Commons, including research sponsors and licensees.

Contract Conflicts I represent that, except as identified below, I have no agreements with or obligations to others that would conflict with my obligations under this Participation Agreement, including but not limited to: sponsored research agreements or technology licenses with terms and conditions incompatible with my participation in the Project; employment or post-employment agreements with present and prior employers other than my institution; consulting contracts; agreements to provide services as a member of a technical or advisory board to a third party; and any other external commitments.

Signature Date

Witness Date

List any and all additional conditions or conflicting agreements and obligations on a separate page.
